# Land use and life history constrain adaptive genetic variation and reduce the capacity for climate change adaptation in turtles

**DOI:** 10.1186/s12864-021-08151-7

**Published:** 2021-11-18

**Authors:** Nathan W. Byer, Emily D. Fountain, Brendan N. Reid, Kristen Miller, Paige J. Kulzer, M. Zachariah Peery

**Affiliations:** 1 University of Nevada–Reno, NV 89557 Reno, USA; 2 University of Wisconsin–Madison, 53706 Madison, WI USA; 3 W.K. Kellogg Biological Station, Michigan State University, MI 49060 Hickory Corners, USA

**Keywords:** Genotype-by-sequencing, Custom amplicon, Landscape genomics, Local adaptation, Reptiles

## Abstract

**Background:**

Rapid anthropogenic climate change will require species to adapt to shifting environmental conditions, with successful adaptation dependent upon current patterns of genetic variation. While landscape genomic approaches allow for exploration of local adaptation in non-model systems, most landscape genomics studies of adaptive capacity are limited to exploratory identification of potentially important functional genes, often without *a priori* expectations as to the gene functions that may be most important for climate change responses. In this study, we integrated targeted sequencing of genes of known function and genotyping of single-nucleotide polymorphisms to examine spatial, environmental, and species-specific patterns of potential local adaptation in two co-occuring turtle species: the Blanding’s turtle (*Emydoidea blandingii*) and the snapping turtle (*Chelydra serpentina*).

**Results:**

We documented divergent patterns of spatial clustering between neutral and putatively adaptive genetic variation in both species. Environmental associations varied among gene regions and between species, with stronger environmental associations detected for genes involved in stress response and for the more specialized Blanding’s turtle. Land cover appeared to be more important than climate in shaping spatial variation in functional genes, indicating that human landscape alterations may affect adaptive capacity important for climate change responses.

**Conclusions:**

Our study provides evidence that responses to climate change will be contingent on species-specific adaptive capacity and past history of exposure to human land cover change.

**Supplementary Information:**

The online version contains supplementary material available at 10.1186/s12864-021-08151-7.

## Background

Climate change will be among the greatest challenges facing biodiversity through the 21st century [1–3]. The intensity and speed of contemporary climate change are likely outside of the range of conditions previously experienced by most species on earth, which will require comparatively rapid adaptive responses by species unable to disperse to favorable climate conditions or with limited plasticity [3–6]. Although evolutionary rescue due to novel mutations will be important for climate change responses [7, 8], current levels of adaptive genetic variation, defined as differences among genomes of individuals resulting from natural selection, may also be important for evolutionary responses [9]. Since the capacity for evolutionary change in the face of environmental change is predicated on sufficient adaptive genetic variation in the present day, characterizing adaptive genetic variation is important for predicting population- and species-level resiliency to climate change [10].

Given that adaptive genetic variation is a function of species-specific traits, adaptive potential is expected to vary among species [9, 11–14]. For example, short-lived species with relatively abbreviated generation times are expected to have a greater capacity to adapt to rapid environmental change than longer-lived species [10]. Furthermore, the likelihood of adaptive responses is also expected to vary along habitat specialist-generalist gradients, with specialists expected to respond via natural selection to environmental change and generalists expected to respond through phenotypic plasticity [15, 16]. Thus, a better understanding of how species-specific characteristics affect adaptation will be crucial for making predictions of climate change responses [10].

Since local adaptation represents an interaction between genetic polymorphisms with functional roles, spatial variation in selection pressures, and phenotypic effects on fitness, focusing on genes of known functional significance allows for tests of ecological hypotheses related to adaptation [9, 10]. Indeed, such studies have proven instrumental for identifying and understanding gene functional groups likely to be important for local adaptation to climate conditions within and among taxa. For example, the gene GIGANTEA-GI5, which has documented interactions with plant circadian clocks [17], has been found to be strongly associated with spatial variation in temperature, providing initial evidence that this gene is involved in local adaptation to temperature [18]. A number of additional gene functional groups – including genes related to stress response [19] and development [20] – hold promise for further study of the capacity for local adaptation to climate change.

Identifying functional genes important for climate change adaptation in non-model organisms often relies on landscape genomic approaches for investigating adaptive genetic variation [9, 21]. Tests of associations between functional genetic variation – defined in this study as genetic variants that potentially affect protein function and expression, and thus may be under selection – and environmental features can be used to explore and identify environmentally-imposed selective pressures that may have shaped adaptive genetic variation [9, 21, 22]. Moreover, characterizing spatial patterns in functionally-relevant gene regions allows for identification of populations that are expected to be less capable of responding adaptively to climate change, which can then be used to prioritize conservation efforts [18, 22]. Finally, functional genes that vary along environmental gradients represent potentially useful targets for more detailed investigations of how functional genetic variation may have consequences for fitness across the landscape [9, 22].

While substantial progress has been made in identifying and exploring local adaptation, several limitations challenge landscape genomic studies. First, most studies do not compare genes of known function to independent, unlinked neutral regions, instead relying on screening large numbers of loci to identify either markers that are more related to environmental gradients or more divergent between populations than others; this relies on the assumption that these outlier loci hold adaptive significance, yet very few outlier loci are often found to match with genes of known function [9, 23]. Second, many landscape genomic studies of local adaptation focus on a single target species, despite evidence that patterns of local adaptation are influenced by species-specific life history traits [10, 24, 25]. The influence of species traits and life history on local adaptation means that insights into the capacity for local adaptation may not transfer among species, which will necessitate studies that delve into the species-level traits that drive local adaptation [9, 10, 25]. These factors collectively limit inferences from many landscape genomic studies of local adaptation to initial, exploratory identification of loci worth further investigation, and challenge the testing of ecological and evolutionary hypotheses.

In this study, we addressed several of the aforementioned limitations by characterizing how putatively neutral and putatively adaptive genetic variation in two turtle species is affected by species-specific life history and habitat utilization traits and environmental characteristics. We addressed questions related to adaptive genetic variation using turtles for several reasons. Turtles, with their long lifespans and delayed sexual maturity, may have a limited capacity to respond rapidly to environmental change [26–28]. However, turtles possess three characteristics directly tied to climatic conditions: (1) they are ectotherms, and many physiological processes are directly linked to thermal characteristics; (2) many species exhibit Temperature-Dependent Sex Determination (TSD) whereby hatchling sex ratios are a function of incubation temperatures [29], and (3) a number of temperature-related traits, including TSD, display documented spatial differentiation in turtles [30]. These three features suggest that increases in temperature may be more likely to lead to adaptive change for turtles than many other taxa, despite life history traits that may reduce the likelihood of adaptive responses. Therefore, landscape genomics tools may provide important insights into whether turtle populations possess sufficient adaptive genetic variation to allow evolutionary response to climate change.

We focused our analysis to two species: the Blanding’s turtle (*Emydoidea blandingii*) and the snapping turtle (*Chelydra serpentina*). The Blanding’s turtle (*E. blandingii*) is a medium-bodied freshwater turtle that requires a juxtaposition of open terrestrial habitat for nesting with permanent wetlands for foraging and overwintering [31]. Due to pressures posed by habitat loss and road mortality, combined with this species’ long generation times (36-47 years), *E. blandingii* is currently listed on the IUCN Red List as “Endangered”, and declines have been observed across much of the species’ range[32]. The snapping turtle (*C. serpentina*) is a large-bodied freshwater generalist that is widely distributed throughout the United States and Canada [31]. Although both species have TSD, in *E. blandingii* females are produced at incubation temperatures higher than a certain pivotal temperature, whereas for *C. serpentina* males are produced at intermediate temperatures and females are produced at extreme temperatures [31]. Importantly, the range of incubation temperatures producing male and female hatchlings exhibits heritable variation [33] and varies spatially across snapping turtle populations [34], although intraspecific variation in TSD has yet to be examined in Blanding’s turtle.

 We used both genome-wide discovery and genotyping of SNPs and targeted sequencing of four target genes that hold potential functional importance for adaptation to climate change at regional and local scales to test several hypotheses. First, we hypothesized that variation in targeted gene regions may be of adaptive importance; thus, we predicted that spatial genetic variation in targeted gene regions should differ from patterns observed with putatively neutral SNP loci. Second, we hypothesized that species-level life and natural history traits should influence putatively adaptive genetic variation. From this hypothesis, we explored two contrasting predictions. One alternative is that habitat specialization leads to a predisposition towards evolutionary adaptation over plastic responses in the face of environmental change [35, 36]; under this prediction, we may expect stronger environmental associations and more putatively adaptive genetic variation in the habitat specialist, *E. blandingii*, than the generalist, *C. serpentina.* In contrast, in our system, the sensitive conservation status of the Blanding’s turtle and its lower effective population sizes may mean that it has less adaptive genetic variation overall and weaker environmental associations than the widespread *C. serpentina*. Finally, we hypothesized that environmental associations in targeted genes should reflect the putative functions fulfilled by each gene. From this we predicted stronger associations between climatic conditions than land cover in genes associated with temperature-sensing and sex determination; in contrast, we predicted associations with both land cover and climate variables in genes related to stress response. By addressing evolutionarily- and ecologically-relevant hypotheses about local adaptation with multiple lines of genetic evidence, we provide novel insights into how species-level traits and environmental features interact to constrain local adaptation.

## Results

### SNP statistics

The *E. blandingii* SNP dataset consisted of 2,811 SNPs across 37 individuals, and had a mean minor allele frequency (MAF) of 0.210, a mean *H*_*O*_ of 0.192, and a mean *H*_*E*_ of 0.294. The *E. blandingii* SNP dataset global *F*_*ST*_ was 0.0300, and 20/21 pairwise comparisons between sites were significant despite low sample sizes (95.2 %; Supplementary Materials S1). For these SNPs, we detected a significant effect of isolation-by-distance based on a Mantel test of individual genetic and geographic distances (*r* = 0.306, *p* = 0.015). The *C. serpentina* SNP dataset consisted of 1,606 SNPs across 33 individuals, and had a mean MAF of 0.169, a mean *H*_*O*_ of 0.169, and a mean *H*_*E*_ of 0.244. Global *F*_*ST*_ was 0.068, and 9/10 pairwise comparisons between sites were significant despite low sample sizes (90 %; Supplementary Materials S1). For these SNPs, we detected a significant effect of isolation-by-distance based on a Mantel test of individual genetic and geographic distances (*r* = 0.363, *p* = 0.001).

### Targeted gene statistics

For *E. blandingii*, a total of two variant sites were identified for CIRBP, six for HSPA8, one for Sox9, and one for TRPV1; after excluding variant sites with the minor allele represented in fewer than two individuals for the unfiltered dataset, this left two for CIRBP, three for HSPA8, zero for Sox9, and one for TRPV1 (Table 1). Although most variant sites were synonymous or noncoding, both polymorphisms for CIRBP coded for amino acid changes (Supplementary Materials S2). Genetic diversity was generally low (*H*_*O*_ = 0.0795), but was highest for one of three variants discovered in HSPA8 (0.278). Across all target genes and sample sites, global *F*_*ST*_ was lower than global *F*_*ST*_ for the SNP dataset (0.0246), and 33/120 pairwise comparisons between sample sites were significant (27.5 %; Supplementary Materials S3). We failed to detect a significant effect of isolation by distance as well, based on a Mantel test of individual genetic and geographic distances (*r* = 0.107, *p* = 0.097).

**Table 1:** Variant sites (with minor allele present in at least 2 individuals upon initial filtering) per species and target gene region. EB = Blanding’s turtle (*Emydoidea blandingii*), and CS = snapping turtle (*Chelydra serpentina*)

Gene	Species	# variants
CIRBP	EB	2
HSPA8	EB	3
Sox9	EB	0
TRPV1	EB	1
CIRBP	CS	0
HSPA8	CS	6
Sox9	CS	0
TRPV1	CS	9

We observed more variants in target genes overall for *C. serpentina*, with zero variant sites for CIRBP, six for HSPA8, zero for Sox9, and nine for TRPV1; however, the nine variant sites in TRPV1 characterized only two distinct haplotypes, and were thus analyzed as one distinct marker (Table 1). Although most variant sites were synonymous or noncoding, three variants for TRPV1 coded for amino acid changes (Supplementary Materials S2). Mean *H*_*O*_ was 0.129, global *F*_*ST*_ was low er than *F*_*ST*_ for the SNP dataset for target genes (0.0590), and only 18/91 pairwise comparisons between sites were significant (18.6 %; Supplementary Materials S3). We detected a significant effect of isolation by distance as well (*r* = 0.070, *p* = 0.03).

### Spatial patterns and clustering

**Fig. 1:**
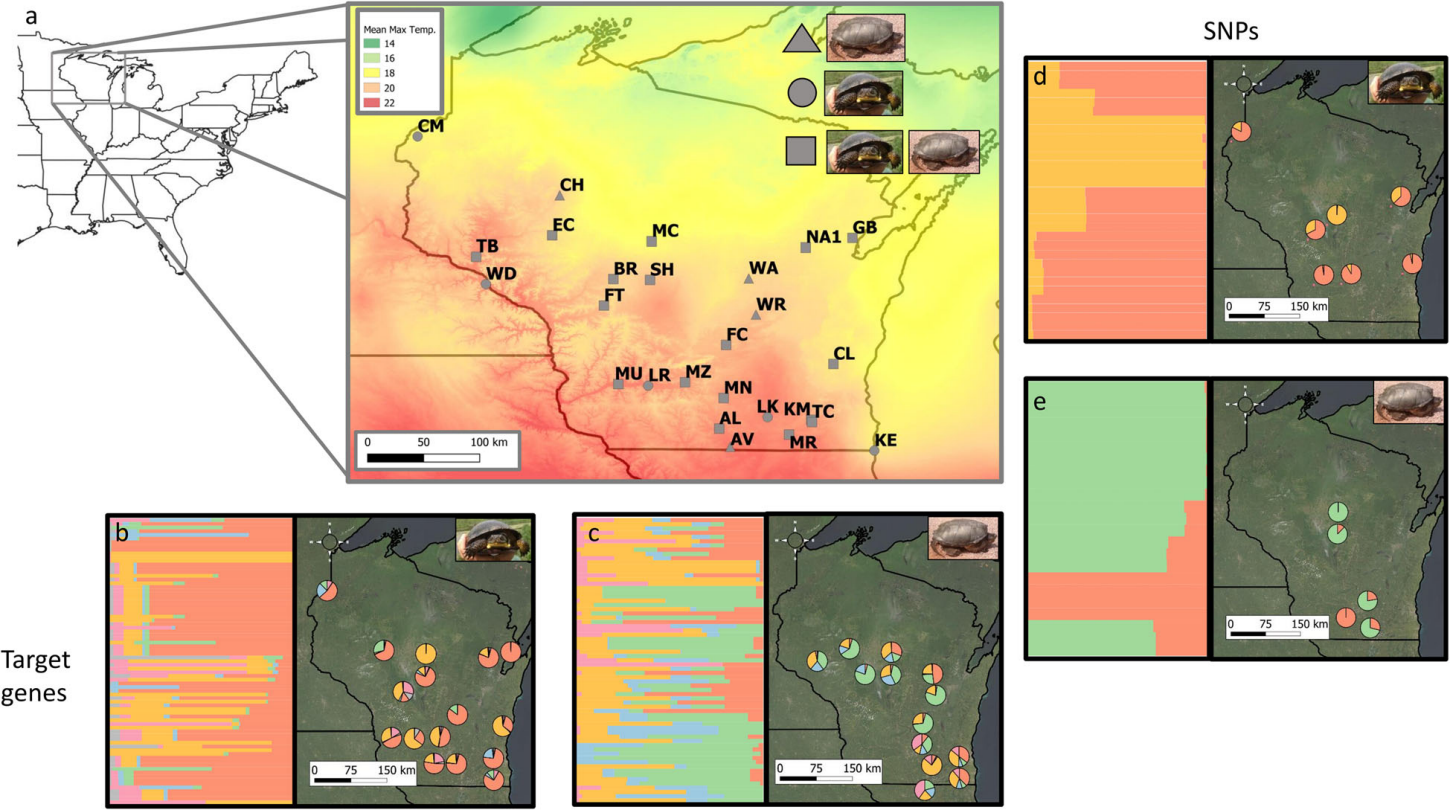
**a** Location of study area and sampling locations in Wisconsin, with each site assigned an alphanumeric code. Color gradients depict variation in mean monthly maximum temperatures, and symbol shapes designate sites where either just Blanding’s turtles (circles), just snapping turtles (triangles), or both species (squares) were sampled. **b**-**c** Admixture bar plots (left) and maps of primary genetic clusters (right) for the *E. blandingii* target gene (**b**) and SNP (**c**) datasets. **d**-**e** Admixture bar plots (left) and maps of primary genetic clusters (right) for the *C. serpentina* target gene (**d**) and SNP (**e**) datasets

The number of genetic clusters and their distribution on the landscape differed between clustering approaches, datasets, and species. For the *E. blandingii* target dataset, the number of inferred clusters varied between approaches, with *K* = 4 for DAPC and *K* = 6 for TESS3 based on observed inflection points in cross-validation scores; in contrast, the number of inferred clusters for the *E. blandingii* SNP dataset was lower, ranging from *K* = 1 for DAPC to *K* = 2 for TESS3, with no clear inflection points observed in cross-validation scores (Supplementary Materials S4). Although clusters for both datasets largely overlapped spatially, these clustering approaches did highlight sites that contained ancestry from relatively few clusters for target genes (FC, GB, and MC) and sites that represented more inferred clusters (CM, FT, MU, and SH, Fig. 1). For the *C. serpentina* target dataset, the number of inferred clusters varied from *K* = 3 for DAPC to *K* = 5 for TESS3; in contrast, the number of inferred clusters for the associated SNP dataset was lower, with *K* = 1 for DAPC and *K* = 2 for TESS3 (Fig. 1; Supplementary Materials S4). As with the *E. blandingii* SNP dataset, we observed no clear inflection points in cross-validation scores for the *C. serpentina* SNP dataset.

### Species- and dataset-level environmental associations

**Fig. 2:**
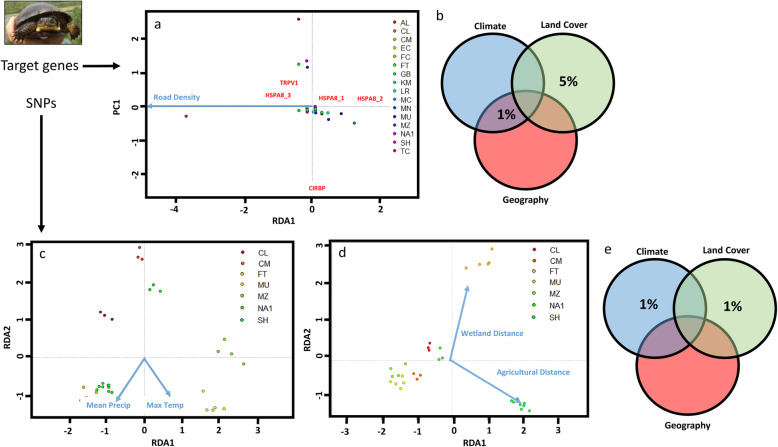
**a** Best-supported partial redundancy analysis (pRDA) for the *E. blandingii* target dataset, depicting the relationship between environmental axes and the first axis of genetic differentiation (PC1). Blue arrows indicate supported environmental relationships comprising redundancy axes; note that the best-supported pRDA included only a single predictor variable (road density) represented along the first constrained axis (RDA1). Colored points indicate loadings of samples from each sample site in multivariate space, and interposed red text indicates loadings of each identified marker for CIRBP, HSPA8 (HSPA8_1, HSPA8_2, HSPA8_3), and TRPV1 in multivariate space. **b** Variance partitioning between climate, land cover, and geography for the *E. blandingii* target dataset, indicating higher percent variance explained for the land cover dataset. Overlapping regions between circles indicate variance partitions correlated across variable classes. **c**-**d** best supported pRDA for the relationship between climate variables (**c**) or land cover variables (**d**) and genetic variation in the *E. blandingii* SNP dataset, with coloring of lines and symbols as described above. **e** Variance partitioning between climate, land cover, and geography for the *E. blandingii* SNP dataset

Although we detected more nonsynonymous substitutions for *C. serpentina* than *E. blandingii*, stronger environmental associations for the *E. blandingii* target dataset indicated support for the prediction that habitat specialists are more likely to be locally adapted to environmental conditions. For the *E. blandingii* target dataset, we detected a significant association between genetic differentiation and road density (*F* = 3.503, *p* = 0.042; Fig. 2), and variance partitioning indicated that the largest proportion of variance was explained by land cover covariates (5 %; Fig. 2). In contrast, for the *E. blandingii* SNP dataset, while we detected significant associations with distance to agricultural cover (*F* = 1.2187, *p* = 0.001), distance to wetlands (*F* = 1.1459, *p* = 0.004), precipitation (*F* = 1.1272, *p* = 0.006), and maximum temperature (*F* = 1.1920, *p* = 0.001), variance partitioning revealed that both climate and land cover covariates explain very little variance overall (about 1 % of total variance each; Fig. 2). For the *C. serpentina* target dataset, we failed to detect any significant environmental associations, and variance partitioning indicated that less than 1 % of total variance was explained by climate, land cover, and geography covariates. In addition, we failed to detect any significant associations between climate or land cover and genetic variation in the SNP dataset, although proportions of variance explained were approximately equal across climate, land cover, and geography (3-4 %; Supplementary Materials S5).

### Gene-specific environmental associations

**Fig. 3:**
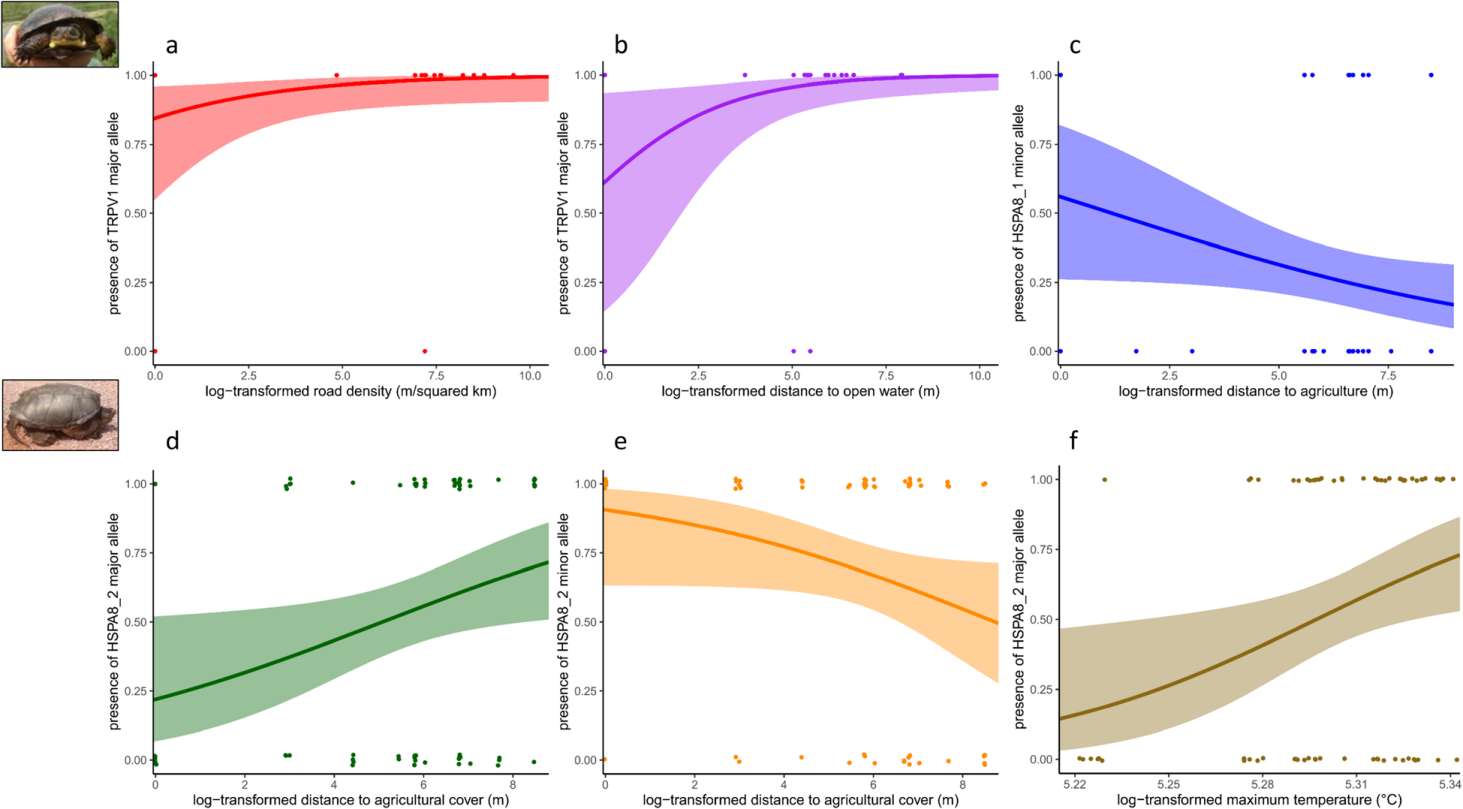
**a**-**c** Plots of relationships between allele presence/absence and environmental features for *E. blandingii*. **d**-**f** Plots of relationships between allele presence/absence and environmental features for *C. serpentina*. Colored lines indicate predicted relationships, shaded areas indicate confidence intervals, and colored points indicate observed data points for corresponding colored lines

For the *E. blandingii* target gene dataset univariate analyses, likelihood ratio tests identified three univariate logistic regression models of allele presence/absence that outperformed null expectations. First, a model of presence/absence of the TRPV1 major allele against road density was significant (*χ*^*2*^ = 4.0555, *p* = 0.044), and indicated that presence of the major allele was associated with higher log-transformed road densities (*β*_roads_ = 0.3302, 95 % CI = 0.009 to 0.7599, Fig. 3). Second, a model of presence/absence of the TRPV1 major allele against distance to open water was significant (*χ*^*2*^ = 4.8221, *p* = 0.028), and indicated that presence of the major allele was associated with greater distance to open water (*β*_open_ = 0.5266, 95 % CI = 0.0627 to 1.0156, Fig. 3). Third, a model of presence/absence of the minor allele for the first HSPA8 variant against distance to agricultural cover was significant (*χ*^*2*^ = 4.0168, *p* = 0.045), and indicated that presence of the minor allele was associated with reduced distances to agricultural cover (Fig. 3). For gene-specific multivariate partial redundancy analyses conditioned on latitude and longitude, we identified only a significant association between genetic differentiation in HSPA8 and road density (*F* = 6.8965, *p* = 0.011; Supplementary Materials S6).

In contrast, for the *C. serpentina* target gene dataset, likelihood ratio tests identified three models of allele presence/absence that outperformed null expectations, all associated with the second documented HSPA8 variant site. First, models of presence/absence of the minor allele for the second HSPA8 variant site against maximum temperature (*χ*^*2*^ = 6.7824, *p* = 0.009206) and distance to agricultural cover (*χ*^*2*^ = 5.272, *p* = 0.02167) were both significant, and indicated positive associations with both maximum temperature (*β*_tmax_ = 21.746, 95 % CI = 5.106 to 41.554) and distance to agricultural cover (*β*_*ag*_ = 0.2494, 95 % CI = 0.0352 to 0.4962) and allele presence. Finally, a model of presence/absence of the major allele of the second HSPA8 variant site against distance to agricultural cover was significant, and indicated a negative association between presence of this allele and distance to agricultural cover (*β*_*ag*_ = -0.2593, 95 % CI = -0.5672 to -0.0107). For gene-specific multivariate partial redundancy analyses conditioned on latitude and longitude, we failed to detect any significant associations between climate or land cover variables and genetic differentiation.

## Discussion

In this study, we leveraged both genome-wide SNPs and targeted sequencing of genes potentially important for local adaptation to explore how life history affects patterns of local adaptation. By exploring potentially adaptive genetic variation in a widespread generalist with an earlier age of maturity (*C. serpentina*) and a more range-restricted specialist with substantially delayed maturity (*E. blandingii*), we explored the genetic consequences of life history traits and habitat specialization [15, 16]. Moreover, we documented sample site-specific polymorphisms in most target genes examined. Several of these polymorphisms were nonsynonymous, and may have adaptive significance (Supplementary Materials S2). While many of these polymorphisms did not lead to changes in gene function, polymorphisms in non-coding regions may still be under positive selection and may still play a role in functional gene regulation [37]. By exploring how variation in these polymorphisms differed from variation in putatively neutral SNPs across a regional extent, we provided initial evidence that spatial heterogeneity in selective pressure associated with environmental conditions shaped among-population variation in these target genes.

### Spatial patterns in neutral and adaptive genetic variation

Neutral and adaptive genetic variation are expected to be shaped by different processes, which should produce contrasting representations of spatial clustering between putatively neutral and adaptive genetic markers [24, 38, 39]. As expected, the numbers and spatial arrangement of identified genetic clusters differed between putatively neutral SNPs and target genes (Fig. 1). In particular, although we failed to observe clear inflection points in cross-validation scores for both SNP datasets, clusters identified for these datasets always corresponded to cohesive spatial groupings. For the *C. serpentina* SNP dataset, site AL represented a separate cluster from all other sample sites; in addition, other sites in the southern portion of the state (sites AV and MN, Fig. 1) had greater representation of this cluster as well. For the *E. blandingii* SNP dataset, site SH – one of the largest and best-studied Blanding’s turtle populations in Wisconsin [40–43] – was assigned a unique cluster, highlighting the importance of protecting this site in the state. In contrast, the target gene panel was classified into a larger number of clusters that: (a) did not appear to correspond to clusters observed in the SNP datasets, and (b) overlapped spatially.

Although this appears to support our hypothesis – that spatial patterns in genetic differentiation in target gene and SNP datasets should reflect contrasting processes governing adaptive and neutral genetic variation, respectively – there are several caveats to these findings. First, although our intention for sequencing these particular gene regions was to target regions of potential adaptive importance, the limited number of genomic regions targeted by this approach relative to genotype-by-sequencing (four versus several thousand, respectively) may have produced differences in spatial genetic structure purely due to sampling effects, rather than effects of ecological and evolutionary processes. Second, any clustering in target gene datasets incorporated variant sites identified across all genes, as low numbers of variant sites identified overall made gene-specific clustering intractable. Given that we observed differences in environmental associations in both gene and allele-level analyses (see below), the spatial overlap in target gene clusters likely indicates that different processes are producing patterns in each gene region. Third, low sample sizes per site likely led to incomplete depictions of spatial patterns in allele frequencies. Although we attempted to use approaches throughout that do not have documented biases at low site-level sample sizes, clustering algorithms are affected by choices in sampling strategy [44]. Despite these caveats, these findings do suggest that highly divergent spatial genetic patterns may be observed between gene-specific and genome-wide datasets, and provide justification for further investigations into environmental associations.

### Species-specific environmental associations

In addition to documenting divergent patterns of spatial clustering across datasets, we also documented significant environmental associations with adaptive genetic variation, suggesting that environmental heterogeneity may exert selective pressure on these genetic regions [9, 14]. Furthermore, our comparison of two species at opposite ends of a habitat specialist-generalist gradient allowed for consideration of how habitat specialization affects local adaptation. Although habitat specialists are expected to respond adaptively to climate change [15, 16], habitat specialists often persist in smaller populations than generalists, and small populations are generally expected to have reduced capacity to respond adaptively due to inbreeding effects on fitness and lower expected rates of mutations per unit time [36, 45, 46]. Our habitat specialist species, *E. blandingii*, requires co-occurrence of undisturbed wetlands habitat for residency and upland sandy bluffs for nesting [26, 31, 47], and many populations throughout Wisconsin are expected to be declining [42, 48, 49]; furthermore, the capacity for local adaptation in this species has been uncertain until now.

We considered three criteria when evaluating the potential effects of habitat specialization on local adaptation: (a) the number of nonsynonymous substitutions in each species-specific dataset, (b) the relative explanatory power of environmental variables for target vs. SNP datasets for each species, and (c) the relative explanatory power of environmental variables for target gene datasets across species. Below, we discuss each of these criteria in turn. First, we documented similar numbers of nonsynonymous substitutions for each species (three in *C. serpentina* and two in *E. blandingii*); furthermore, these nonsynonymous substitutions were observed in different genes across both species, with all three in *C. serpentina* observed in TRPV1 and both in *E. blandingii* in CIRBP. In contrast, climate and land cover covariates explained more variance in the *E. blandingii* target gene dataset than the SNP dataset (5 % vs. <1 %, Fig. 2) and the *C. serpentina* target gene dataset (5 % vs. 0 %; Supplementary Materials S5).

While diversity in these gene regions may facilitate adaptive responses to climate change, the predominance of land cover associations in these target gene datasets may indicate that spatial patterns in diversity for these gene regions has largely been shaped by anthropogenic habitat alterations – which have likely reduced effective population sizes, connectivity, and genetic diversity for both of these species [48, 50, 51] (Fig. 3). Although we expected to document climate associations for both species, we failed to detect any significant climate associations in *C. serpentina* for any genes, and land cover covariates (particularly road density) explained more genetic variation in target genes than climate covariates for *E. blandingii* (Fig. 2). Areas of increased road density have previously been found to cause reduced genetic diversity and biased sex ratios in *E. blandingii*, likely due to increased road mortality risk in areas with more roads [48]. Assuming that these target gene regions represent a reasonable proxy for adaptive genetic variation, our results thus indicate that these same landscape features that reduce neutral genetic diversity and negatively influence population demographics may also affect adaptive potential for this species. Given how many *E. blandingii* populations are located within a matrix of anthropogenic habitat alterations, efforts to protect habitats that are relatively isolated from human activities and reduce or mitigate the effects of roads near critical habitat areas may represent effective strategies for increasing population sizes and maximizing adaptive potential.

### Gene-specific environmental associations

Environmental associations also varied among gene functional categories. First, since both species exhibit temperature-dependent sex determination and prior studies have suggested that plastic nesting behaviors may be insufficient to demographically buffer turtle populations against climate change [52, 53], we predicted that variation in two genes associated with sex determination (CIRBP and Sox9) would be related to environmental conditions. Although both polymorphisms in CIRBP for *E. blandingii* were nonsynonymous (Supplementary Materials S2), neither of these polymorphisms were associated with any environmental gradients, potentially indicating no adaptive divergence in CIRBP due to environmental factors. This is consistent with prior studies that indicate that TSD is a polygenic trait with relatively little heritable variability in wild populations [33, 54, 55]. Furthermore, within-population variation in several traits associated with TSD (sex ratio and transitional range of incubation temperatures, or TRTs) is often much higher than among-population variation [30]. When interpreted with relatively low levels of standing genetic variation documented in our study, this indicates that substantial physiological plasticity in TSD will be more important than standing genetic variation in gene regions associated with TSD for adequate climate responses.

Since such plasticity would depend upon adequate sensing and response to increasing temperatures and TRPV1 has functional associations with temperature-sensing [46], we expected variation in this gene to fall along similar environmental gradients to CIRBP and Sox9. As before, we failed to detect significant associations with climate for TRPV1 in either *E. blandingii* or *C. serpentina*. However, for *E. blandingii*, we did document significant associations between allele presence and land cover covariates (road density and distance to open water). As with the species-specific environmental association analyses, this may indicate that genetic variation in genes critical for adequately adapting to climate change is in some ways decoupled from contemporary climate conditions, and is instead shaped by the demographic and genetic impacts of anthropogenic habitat alterations [48].

Unlike sex-determining genes, we documented more polymorphisms and stronger environmental associations in our stress response gene (HSPA8) than expected. While several previous studies have suggested the importance of heat shock proteins and other genes associated with stress response for screens of local adaptation, little is known about the environmental factors that might select for particular polymorphisms in heat shock proteins [19, 56–59]. Since HSPA8 polymorphisms were associated with road density in *E. blandingii*, anthropogenic landscape alterations may have exerted selective pressures upon this gene [57, 60, 61]. Although physiological stress responses are often not considered in climate change vulnerability assessments, temperature increases through the 21st century will likely exceed the thermal tolerance limits of many species [56]. Given how many heat shock proteins exist further study is needed to determine which heat shock proteins may be implicated in local adaptation, and whether these genes may play a role in responses to environmental change [56, 57, 60, 61]. Although further investigation of the gene region targeted by our HSPA8 primer set indicated that these polymorphisms may be in a noncoding region (Supplementary Materials S2), numerous studies have suggested that adaptation may still be facilitated by variation in noncoding regions [62, 63]. Although it is often unclear if intronic polymorphisms are themselves functional or if they are simply within linkage disequilibrium with unidentified functional polymorphisms potentially in exonic regions, it appears that exploration of noncoding polymorphisms within gene regions may still provide insights into adaptation [64].

## Conclusions

In summary, we leveraged one of the many strengths of landscape genomic approaches – the testing of evolutionarily-relevant hypotheses in field systems– to explore the impacts of habitat specialization and gene function on patterns of putatively adaptive genetic variation [23]. While our conclusions were limited by the lack of additional genomic resources for either species, our study points to several gene regions – in particular HSPA8 and TRPV1 – that may represent promising targets for further, targeted investigations of spatial patterns of local adaptation in these and other non-model organisms [11–13, 23, 65]. As annotated genomes in non-model organisms become increasingly available, the genomic context of functional genes can then be considered in more detail, allowing studies of the genomics of local adaptation to move beyond initial exploratory analyses and towards causal, evolutionarily-grounded investigations of the likelihood of climate change adaptation [23].

Given the scope of conservation challenges posed by climate change, landscape genomic studies will increasingly be leveraged to provide insights into effective conservation and wildlife management [66–68]. In particular, such may facilitate more effective spatial area prioritization and climate change vulnerability assessments for sensitive taxa. In our study, observed differences in the distribution of adaptive and neutral genetic variation mean that spatial prioritization based solely on neutral markers is unlikely to capture the full spectrum of adaptive genetic variation in either species [69, 70]. As with many other landscape genomic studies, however, we were not able to connect genetic variation in these functional genes to phenotypic differences or fitness consequences [38, 71]. As a result, although we were able to identify populations with unique adaptive potential, we do not know which variants in any of these genes confer increased resiliency to environmental change. Although the actual functional significance of spatial variation in our chosen target genes is currently unknown for these two species, prioritizing sites that cover the full range of potentially adaptive genetic variation may maintain the potential for evolutionary adaptation [69, 72]. In the absence of information on how fitness is associated with spatial genetic variation in any of these genes, ecological studies linking phenotypic and genotypic variation in these functional traits to fitness outcomes may provide additional evidence for local adaptation [13, 65, 73].

Studies of local adaptation provide additional advantages beyond spatial prioritization as well, particularly if significant environmental associations with adaptive genetic variation are detected, as these environmental associations may provide evidence of the selective forces underpinning the distribution of adaptive genetic variation [9, 13]. For our specialist species (*E. blandingii*), potentially adaptive genetic variation was more strongly associated with land cover than climate, with particularly strong effects of road density. Given previous findings that roads have demographic and genetic impacts on turtle populations, this may indicate an additional threat posed to turtles by roads, whereby roads reduce the potential for local adaptation [48, 50, 51] However, we caution that adaptation in each of these species may take many generations; thus, associations between potentially adaptive genetic variability and contemporary land cover, climate, and geographical characteristics may not represent conditions that shaped historical adaptive responses [9, 14]. Nonetheless, associations between contemporary patterns of potentially adaptive genetic variation and environmental heterogeneity provide insights into whether adaptive genetic variation is currently distributed along environmental gradients expected to drive selection in the future [9, 11–13]. Information on these potentially important environmental gradients – when combined with effective spatial prioritization – will likely prove to be critical for predicting the role of local adaptation in species persistence in a changing world.

## Methods

### Study species and sample selection

Blanding’s turtles and snapping turtles were sampled across more than 20 sites in Wisconsin between 2010 and 2015 [41, 48, 74] (Fig. 1). Turtles were trapped using a variety of approaches, including hoop traps, crayfish traps, minnow traps, and hand captures. For each captured turtle, we obtained blood samples from the dorsal coccygeal vein, and animals were released following individual marking. We also opportunistically collected tissue samples from museum specimens, road-killed animals, and captive-reared juveniles from egg clutches with known localities [41]. Genomic DNA was extracted from samples using a Qiagen DNeasy Blood and Tissue Kit, as described elsewhere [41, 48]. We subsampled sites and samples from this larger sample set for two separate sequencing efforts: (a) SNP discovery and genotyping and (b) investigation of variation in four functional genes. We selected samples to maximize: (a) variation in climatic conditions across sample sites, as indicated by Worldclim climate data layers [75], and (b) individuals sampled per site. We then randomly selected 3 or more individuals for each species from each site across these sample sites; if a selected site has fewer than 3 individuals for a given species, we used all samples instead (see Table 2 for sample sizes for each species and dataset). For SNP discovery and genotyping, we also only used samples with DNA concentrations suitable for sequencing (>20 ng/uL). While we initially intended to use the same samples for both functional gene and SNP analyses, stringent concentration requirements for the SNP discovery component of the project and low sample volumes meant that some samples were used solely for SNP discovery. Final sample sets were: 50 *C. serpentina* across 16 sites for SNP genotyping, 86 *C. serpentina* across 21 sites for target gene sequencing, 60 *E. blandingii* across 13 sites for SNP genotyping, and 95 *E. blandingii* across 22 sites for target gene sequencing.

**Table 2:** Final sample sizes for each species, dataset, and site

Population	# EB samples		# CS samples	
	Target	SNP	Target	SNP
AL	4	0	0	4
AV	0	0	3	3
BR	0	0	4	0
CH	0	0	0	0
CL	3	3	0	0
CM	3	3	0	0
EC	3	0	4	0
FC	3	0	4	0
FT	7	5	0	0
GB	3	0	0	0
KM	3	0	6	0
LR	3	0	0	0
MC	3	0	7	10
MN	4	0	3	3
MR	0	0	6	0
MU	6	5	4	0
MZ	4	4	0	0
NA	5	3	0	0
SH	20	8	7	3
TB	0	0	6	0
TC	3	0	4	0
WA	0	0	4	0
WR	0	0	5	0

### SNP discovery and genotyping

We used genotype-by-sequencing (GBS) to discover and genotype SNPs for each species (Elshire et al. 2011). Samples were submitted for library preparation and sequencing to the University of Wisconsin-Madison Biotechnology Center. Each sample was digested using apeKI, indexed and then sequenced on an Illumina HiSeq2500. Two libraries of the same 87 samples (51 *E. blandingii*, 36 *C. serpentina)* were single-end 100 bp sequenced in their own lanes; given poor overlap in loci among individuals for these libraries, an additional library of 87 samples (containing 37/51 previously sequenced *E. blandingii* and 27/36 previously sequenced *C. serpentina* samples, with an additional 9 *E. blandingii* and 14 *C. serpentina* samples unique to this library) was also pair-end 100 bp sequenced on a RAPID run in two lanes. Since genomes were not yet available for either species at time of analysis, we then used *de novo* assembly in iPyrad [73] to call SNPs and genotype individuals for these SNPs. Before assembly, we merged raw sequencing output per individual across runs. We outline the iPyrad assembly steps in brief below, which were applied to each species separately. Step 1 involved demultiplexing input fastq files; for this step, we set max_barcode_mismatch to 0 but maintained most other default settings. Step 2 involved filtering and editing the demultiplexed data; for this step, we used the following settings: max_low_qual_bases = 5, filter_adapters = 2, filter_min_trim_len = 35, trim_reads = 0, and Phred_Qscore_offset = 33. Step 3 involved clustering and mapping demultiplexed and filtered reads; we set a clust_threshold of 0.85 for this step. Step 4 involved joint estimation of heterozygosity and sequencing error rates; we set max_alleles_consens to 2 for this step. Given that choices of parameter values for minimum cluster depth (mindepth_statistical and mindepth_majrule) has downstream influences on the number and quality of final SNPs called and genotyped, we tested several parameter sets that maximized both the number of individuals and loci retained and the quality of the final SNP datasets. During this parameter optimization we discovered that most clusters (>70 %) were at coverage levels of 2–4; thus, we ultimately set mindepth_majrule to 5 and mindepth_stat to 10. We also set maxdepth to 10,000 for this step. Step 5 involved consensus base calling and filtering; we set max_Ns_consens to 5 for this step. Step 6 involved clustering and mapping reads across samples and alignment of clustered sequences; we used default settings for this step. Finally, step 7 involved final filtering and creation of output files. For this step, we tried several parameter values for min_samples_locus initially ranging from 5 to 60, but discovered high levels of individual-level missing data as a result of this optimization procedure; thus, we opted to use a low value for min_samples_locus of 5 to facilitate downstream filtering [76]. Other parameter values were as follows: max_Indels_locus = 8, max_shared_Hs_locus = 8, and trim_loci = 0.

Final SNP datasets for each species were exported as vcf files for further filtering using package “dartR” [77]. First, we filtered out monomorphs using “gl.filter.monomorphs”. We then excluded any SNPs with MAF less than 0.05. Next, in keeping with [76], we iteratively and alternately filtered call rates at individual and locus-level using “gl.filter.callrate”, starting with a threshold tolerance for missing data of 0.5 for loci and 0.1 for individuals and ending with a threshold of 0.7 for loci and 0.5 for individuals; this progression was chosen to mimic FS 5 and 6 from [76]. We then used “gl.filter.secondaries” to select one SNP per fragment with the least missing data. We then used “gl.report.hwe” to identify SNPs that deviated from Hardy-Weinberg Equilibrium across all sites, and removed these SNPs using “gl.drop.loc”. Finally, we filtered for heterozygote excess by first calculated observed heterozygosity (*H*_*O*_) for each locus using “gl.basic.stats”, and then used “gl.drop.loc” to drop any loci with *H*_*O*_ > 0.5. The *E. blandingii* dataset comprised 557,829 SNPs across 64 individuals and 12 sites, represented at a mean coverage of 28.58x, each of which was present in at least four individuals. After filtering for MAF, missing data by individual and locus, Hardy-Weinberg Equilibrium, heterozygote excess, and for a single SNP per RAD locus, a total of 2,811 SNPs across 37 individuals and 11 sites were available for *E. blandingii*, with a total missing data percentage of 18.9 %. Excluding sites with fewer than three samples produced a final dataset of 2,810 SNPs across 31 individuals in seven sites. The *C. serpentina* dataset comprised 795,296 SNPs across 52 individuals and 15 sites, represented at mean coverage of 25.25X, each of which was present in at least four individuals. After filtering for MAF, missing data by individual and locus, Hardy-Weinberg Equilibrium, heterozygote excess, and for a single SNP per RAD locus, a total of 1,606 SNPs across 33 individuals and 13 sites were available for *C. serpentina*, with a total missing data percentage of 17.23 %. Excluding sites with fewer than three samples produced a final dataset of 1,600 SNPs across 23 individuals in five sites. Hereafter, we refer to these final genome-wide datasets as our SNP datasets. Given low sample sizes and limited spatial representation of sample sites, we primarily used these datasets as a point of comparison with the targeted sequencing efforts described below.

### Amplification of targeted functional genes

We searched NCBI GenBank [78] for literature-supported functional genes that: (1) play a role in thermoregulatory behaviors, such as genes associated with heat- or cold-sensing; (2) code for heat shock proteins, as these have been shown to correlate with stress related to climatic extremes [60, 61]; and (3) play some role in temperature-dependent sex determination. We then selected genes that have a high degree of literature support for putative functional roles. Given (a) the lack of functional genomic information for either species and (b) the need to develop and optimize novel primers for each selected gene, we limited our inquiry to four genes total: CIRBP, a gene that plays a role with temperature-dependent sex determination [79]; Sox9, a frequently studied gene region implicated in sex determination [80, 81]; HSPA8, a heat-shock protein associated with stress response [82] ; and one Transient Receptor Potential Cation Channel (TRPV1) which has been implicated in temperature-sensing [83]. We downloaded available coding sequences for turtles for each gene from GenBank (Supplementary Materials S1), and imported these sequences into MEGA7 [84]. We then used the alignment algorithm MUSCLE [85] to align sequences across taxa using default settings. We then exported these alignments to Primaclade to identify potential primer sets to use for each gene, and selected primers that maximized the number of variant sites within each amplicon of a target size of 250 bp [86]; given the relative scarcity of functional genomic information for these species and rather than subdividing effort between multiple poorly known genomic regions per gene, we opted to investigate only the 250 bp region within each gene with the most variant sites. Furthermore, although we attempted to target only coding regions for selected genes, the lack of available genomic information made delineation of intron and exon boundaries difficult *a priori*; as a result, some primers inadvertently targeted non-coding sequences (Supplementary Materials S7). However, these non-coding regions may still be under positive selection or play a role in functional gene regulation, and likely still reflect patterns in adjacent coding regions to some degree [37].

We tagged the primers for each of these five primer sets (CIRBP, HSPA8, Sox9, and TRPV1) with Illumina TruSeq Universal adapters for downstream Illumina MiSeq sequencing. We then amplified each tagged primer set for each sample using Qiagen Taq PCR core kits and Phusion High-Fidelity DNA Polymerase kits. See supplementary materials S1 for PCR reactions and parameters. We cleaned amplified product using Ampure XP beads, and quantified DNA concentrations for cleaned product using a Qubit fluorometer. Amplified product was then combined in equimolar proportions for each sample, and custom i5 and i7 indices with stem adapters attached were attached using KAPA HiFi HotStart ReadyMix. The indexed samples were then cleaned with Ampure XP beads to remove unincorporated adapters, quantified and combined into two libraries of 96 samples. Libraries were then sequenced on two Illumina MiSeq 2 × 250 nano runs by the University of Wisconsin-Madison Biotechnology Center. All unique gene sequences will be available online (see supplementary materials S2).

### Functional gene amplicon processing

We used QIIME2 [87], a program designed for filtering and processing custom amplicon sequencing output, to filter and separate pooled reads by individual and gene region. First, we used “dada2” to trim and filter demultiplexed sequences and identify unique genetic variants present in each sample [87]. We then trained a naïve Bayes classifier on sequences downloaded from GenBank for each gene to classify genetic variants identified previously into the five amplified genes [87]. Whenever possible, we attempted to use sequences from our two study species; if gene-specific sequences for our species were unavailable, we used other available sequences from turtles instead. We re-ran this procedure with multiple filtering, trimming, and classification confidence settings to identify optimal settings for each gene that: (1) maximize the number of identified genetic variants for each gene, and (2) minimize spurious or incorrectly-classified sequences for each gene. Although this optimization procedure reduced the number of spurious and incorrectly classified sequences, some chimeras, spurious classified sequences, and potential incidences of barcode-hopping were still observed. Thus, we visually inspected each representative sequence and used NCBI BLAST to filter out genetic variants with <60 % query coverage or <80 % identity for the targeted genes. We then excluded individual-level variants with less than certain sequence count thresholds; for CIRBP, Sox9, and TRPV1, we excluded variants for individuals with less than five reads (due to lower representation of these genes in overall libraries), but used a higher threshold of 10 for HSPA8.

The first Illumina MiSeq nano run generated a total of 726,112 sequences of 250 bp each, whereas the second generated a total of 621,932 sequences of 250 bp each. For most genes, aligning forward and reverse reads resulted in relatively high data loss post-filtering; thus, only forward reads were retained for downstream analyses, most of which were either truncated to 200 bp or left unaltered (depending on the gene and species; see supplementary materials S7). Furthermore, HSPA8 was typically over-represented relative to other gene products; however, sufficient reads were retained for each gene for downstream analysis. After excluding samples that failed to sequence reliably, this left 88 individuals across 22 for *E. blandingii* and 79 individuals across 22 sites for *C. serpentina*. After further removing sites with fewer than 3 individuals, this left 77 individuals across 16 sites for *E. blandingii* and 67 individuals across 14 sites for *C. serpentina*.

Genetic variants within each target gene were summarized for each sample and exported to MEGA7 for alignment. The MUSCLE algorithm (using default settings) was used for multiple sequence alignment of all genetic variants across samples, and variant sites present in greater than one individual were identified using MEGA7 and exported to comma-separated value (csv) format. Genotypes were numerically coded based on minor allele frequencies of each polymorphic site for each individual, and converted to a “genind” object using package “adegenet” in R [88]. For all analyses described below, we refer to target gene amplicons as our amplicon dataset, used primarily to represent putatively adaptive genetic variation. In contrast, our final SNP datasets are primarily used to represent neutral genetic variation given low numbers of SNPs per species. Given low sample sizes per site, we have largely chosen to use approaches that lack strict requirements for site-level sample sizes.

### Spatial genetic divergence

In order to address our first hypothesis – that there are differences in spatial genetic structure between target gene and SNP datasets – we quantified population genetic summary statistics and inferred numbers of genetic clusters. First, we quantified expected heterozygosity (*H*_*e*_) and observed heterozygosity (*H*_*o*_) for the SNP and target gene datasets for each sample site and species. We also quantified pairwise hierarchical *F*-statistics between sample sites for each species and dataset using packages “hierfstat”, “pegas”, and “adegenet” based on the Weir and Cockerham (1984) estimator [89–91]; despite low per-site sample sizes, we believe this choice of estimator is appropriate given recent findings that this estimator is relatively robust at small sample sizes, particularly when using more than 1,000 SNP markers [92]. While low numbers of markers for the target gene datasets have likely reduced power for finding statistically-significant population structure, we have retained such analyses to provide a point of comparison with the SNP *F*_*ST*_ calculations. In all cases, we assessed significance using permutation tests with 100 bootstrapped replicates. We tested for isolation by distance for each dataset and species using Mantel tests by relating a matrix of individual-level genetic distances to a matrix of Euclidian geographic distances between samples [93]. While we initially intended to use the SNP datasets to calculate effective population size for each sample site and species, low numbers of genotyped SNPs made such analyses intractable.

In order to identify whether genetic variation was geographically structured among sample sites, we used several approaches to identify genetic clusters in each dataset. First, we used successive *K*-means clustering and discriminant analysis of principal components (DAPC) in package “adegenet” in R to capture spatial clustering in the SNP and amplicon datasets. In order to do so, we used the “find.clusters” function to detect the optimal number of clusters for each species and dataset based on Bayesian Information Criterion (BIC), which generally coincided with a sharp elbow in the curve of BIC values as a function of *K* [37, 86]. We then used DAPC to depict the clusters in multivariate space for each species and dataset. Second, we identified clusters using “tess3r”, a spatially-explicit clustering approach [94]. This approach estimates ancestry coefficients while accounting for information on spatial sample coordinates to control for population structure. As above, we visualized cross-validation scores for each value of *K*, and inferred the optimal *K* based on when cross-validation scores plateau or start increasing [94]. For each algorithm, we tested all values from *K=* 1 to 12.

### Environmental associations

We sampled environmental covariates from a variety of sources, with a similar methodology to that described in [42]. Rather than representing individual sampling locations within each site when sampling environmental conditions, we used a single site-level coordinate for each individual; thus, each individual sampled at a particular sample site is assumed to experience the same environmental conditions. We then extracted climate, land cover, and environmental covariates from a variety of data sources for each sample site. Given that each chosen gene region had functional associations with climate conditions [79, 80, 82, 83], we used WorldClim 1.0 [75] to calculate three climate and weather variables throughout the active season (April to November): mean monthly maximum temperature, mean monthly minimum temperature, and mean monthly precipitation. We calculated distances to several core land cover types critical for population persistence for these species (open water and wetland cover; [31]) or known to reduce gene flow between turtle populations (urban and agricultural cover; [48]) using the WISCLAND dataset (https://dnr.wi.gov/maps/ WISCLAND.html) by first converting raster representations of land cover to polygons, and then calculating Euclidian distance from sample sites to each land cover type. Finally, since road density has documented impacts on genetic diversity for *E. blandingii* [48] we extracted road densities (meters/km^2^ pixel) from the TIGER dataset (https://www.census.gov/geo/maps-data/data/tiger.html). We grouped these covariates into three categories: climate/weather (mean maximum and minimum monthly temperatures, mean monthly precipitation), land cover (distance to urban cover, agricultural cover, open water, and wetlands, and road density), and geographic (latitude and longitude).

In order to address our hypothesis that species-level traits will lead to divergent environmental trends in adaptive differentiation, we used similar genotype-environment association tests as described above. First, for each overall dataset (SNP and target gene) and species (*C. serpentina* and *E. blandingii*), we used the variance partitioning function “varpart” in package “vegan” to assess the variance in allele counts explained by climate, land cover, and geographic variables for each genetic variant in each gene. Next, we ran similar pRDA models to those described above with forward and backward stepwise variable selection to select climate and land cover covariates associated with overall genetic variation in each dataset and species, and used permutation tests to assess variable significance. We used output from these tests to assess relative support for alternative interpretations of our hypothesis. If cumulative variance explained by land cover and climate covariates for the *E. blandingii* target dataset (a) has more nonsynonymous substitutions than the *C. serpentina* dataset, (b) exceeds the proportion for the putatively neutral *E. blandingii* SNP dataset and (c) exceeds the proportion explained for the *C. serpentina* target dataset, our prediction that habitat specialists will show stronger adaptive responses to environmental gradients would be considered supported. Alternatively, if the opposite of all three of these conditions is true, then we will interpret that as evidence that the sensitive conservation status and lower effective population sizes of the Blanding’s turtle may lead to a lower potential of adaptive responses to environmental gradients than the widespread *C. serpentina*. Between these two alternatives, mixed evidence in support of either or both predictions may be possible as well, and may illustrate the relative roles of drift and selection in driving adaptive divergence for these species.

In order to test our hypothesis that environmental associations in targeted genes should reflect the putative functions fulfilled by each gene, we used a combination of variance partitioning approaches and multivariate environmental association tests. First, we classified each polymorphism as noncoding or coding based on alignment of amplicon sequences to previously-described GenBank records; if a polymorphism was determined to be in a coding sequence, we then classified polymorphisms as synonymous or non-synonymous using MEGA7. Next, we used a similar variance partitioning approach to the one described above to assess overall gene-specific differences in the proportion of the genetic variation explained by climate, land cover, and geography. Next, we used both univariate and multivariate tests of environmental associations to assess relationships between variants and environmental gradients potentially driving selection. For univariate tests, we used generalized linear models with allele presence/absence as the response and environmental variables as predictor variables. We limited inquiry to one-predictor models to facilitate downstream analysis, and evaluated significance using likelihood ratio tests. Finally, we used partial redundancy analysis (pRDAs) in the “vegan” library to conduct multivariate tests of genotype-environment associations [14, 95]. Before doing so, we used the function “findCorrelation” in package “caret” to identify and drop environmental covariates in each target dataset that were highly correlated (*r* > 0.6). We then used a forward and backward stepwise variable selection procedure in package “vegan” to select covariates associated with genetic variation in target genes while conditioning for geographic coordinates (latitude + longitude), and used permutation tests to assess variable significance [95].

## Supplementary Information

**Additional file 1.**

## Data Availability

The datasets used and/or analysed during the current study are available from the corresponding author on reasonable request.
