# Supplementary material for: Land use and life history constrain adaptive genetic variation and reduce the capacity for climate change adaptation in turtles
Source: BMC Genomics. 2021 Nov 18;22:837. doi: 10.1186/s12864-021-08151-7 (PMC8603537; doi:10.1186/s12864-021-08151-7)
Supplement: Supplementary file 1 — Additional file 1. [file 12864_2021_8151_MOESM1_ESM.pdf]

Supplementary Materials

Title: Land use and life history constrain adaptive genetic variation and reduce the capacity for climate change adaptation in turtles

Authors: Nathan W. Byer, Emily D. Fountain, Brendan N. Reid, Kristen Miller, Paige J. Kulzer, M. Zachariah Peery

Suppelementary Materials S1: Pairwise F_{ST} for the (a) *E. blandingii* and (b) *C. serpentina* SNP datasets.

(a) *E. blandingii*

Population1	Population2	Lower bound CI limit	Upper bound CI limit	p-value	Fst
FT	MZ	0.010767	0.037309	0	0.027446
FT	SH	0.009295	0.027163	0	0.018606
FT	CM	0.007381	0.050837	0	0.028659
FT	MU	0.023056	0.043653	0	0.033804
FT	NA1	-0.00126	0.027435	0.03	0.014236
FT	CL	0.019831	0.056104	0	0.039409
MZ	SH	0.024137	0.047024	0	0.037533
MZ	CM	0.010972	0.060254	0.01	0.036231
MZ	MU	0.001278	0.029928	0.02	0.018008
MZ	NA1	-0.01706	0.025223	0.24	0.007595
MZ	CL	0.016943	0.058083	0	0.041598
SH	CM	0.049111	0.074868	0	0.059884
SH	MU	0.028726	0.044634	0	0.036786
SH	NA1	0.003531	0.020979	0	0.012264
SH	CL	0.033175	0.059171	0	0.047642
CM	MU	0.036782	0.075551	0	0.059225
CM	NA1	0.017114	0.06218	0	0.038023
CM	CL	0.027612	0.086151	0	0.055434
MU	NA1	0.007274	0.03932	0	0.025453
MU	CL	0.041376	0.073428	0	0.058631
NA1	CL	0.009153	0.050505	0	0.032168

(b) *C. serpentina*

Population1	Population2	Lower bound CI limit	Upper bound CI limit	p-value	Fst
MC	AV	0.054366	0.09538	0	0.075301
MC	SH	0.050891	0.099756	0	0.078402
MC	AL	0.115706	0.150303	0	0.134013
MC	MN	0.040421	0.078406	0	0.063339
AV	SH	-0.01721	0.055627	0.15	0.022484
AV	AL	0.070612	0.122111	0	0.094364
AV	MN	0.001712	0.047518	0.02	0.026501
SH	AL	0.133749	0.187865	0	0.162312
SH	MN	0.0381	0.102182	0	0.075214
AL	MN	0.084654	0.125981	0	0.105441

Supplementary materials S2. Genetic variants for each gene and species. Accession numbers will be added at a later date, following uploading to GenBank.

Species	Gene	Polymorphism (major/minor)	Synonymous, if coding?	Position in fragment	Prop. ind w/minor allele	Accession
**CS**	HSPA8	A/C	Noncoding	37	15/80	Pending
		A/C	Noncoding	39	42/80	Pending
		G/A	Noncoding	62	16/80	Pending
		A/C	Noncoding	86	6/80	Pending
		C/T	Noncoding	136	9/80	Pending
		G/A	Noncoding	137	6/80	Pending
	TRPV1	T/C	Synonymous	90	2/80	Pending
		C/T	Synonymous	93	2/80	Pending
		A/G	Glu → Asn	94	2/80	Pending
		C/A	Glu → Asn	96	2/80	Pending
		C/A	Synonymous	108	2/80	Pending
		G/A	Synonymous	120	2/80	Pending
		A/G	Synonymous	201	2/80	Pending
		C/T	Synonymous	204	2/80	Pending
		T/G	Val → Gly	236	2/80	Pending
**EB**	CIRBP	T/C	Val → Ala	92	8/89	Pending
		A/G	Lys → Glu	141	8/89	Pending
	HSPA8	C/T	Noncoding	20	1/89	Pending
		T/C	Noncoding	32	1/89	Pending
		T/A	Noncoding	52	29/89	Pending
		T/C	Noncoding	54	2/89	Pending
		A/G	Noncoding	133	2/89	Pending
		T/C	Noncoding	159	1/89	Pending
	Sox9	T/C	Synonymous	229	2/89	Pending
	TRPV1	T/C	Synonymous	171	8/89	Pending

Supplementary Materials S3: Pairwise F_{ST} for the (a) *E. blandingii* and (b) *C. serpentina* target datasets.

(a) *E. blandingii*

Population1	Population2	Lower bound CI limit	Upper bound CI limit	p-value	Fst
FT	MC	-0.166666667	0.073529	0.56	-0.00584
FT	MU	-0.060091293	-0.02439	1	-0.04741
FT	CL	-0.166666667	-0.00451	1	-0.08929
FT	MZ	-0.098039216	0.02752	0.94	-0.066
FT	NA1	-0.055276382	0.050104	0.88	-0.02369
FT	SH	-0.0119471	0.082745	0.12	0.023362
FT	TC	-0.166666667	0.013812	0.95	-0.06707
FT	AL	-0.1002616	-0.07095	1	-0.08674
FT	EC	-0.142215569	0.04794	0.85	-0.04386
FT	FC	-0.139059619	0.05491	0.67	-0.02525
FT	GB	-0.166666667	0.013812	0.87	-0.05
FT	CM	-0.245844875	-0.02443	0.99	-0.11364
FT	KM	-0.245844875	-0.00451	0.98	-0.10572
FT	LR	-0.129204893	0.057078	0.89	-0.06279
FT	MN	-0.098039216	0.02752	0.94	-0.05855
MC	MU	-0.074074074	NA	0.25	0.13986
MC	CL	-1.11E-16	NA	0.7	-1.11E-16
MC	MZ	0.067961165	NA	0	0.067961
MC	NA1	0.309927361	NA	0	0.309927
MC	SH	-0.091279526	0.279891	0.3	0.205004
MC	TC	-8.33E-17	NA	0.25	0.3
MC	AL	-0.044585987	NA	0.25	0.181556
MC	EC	-8.33E-17	0.5	0.11	0.25
MC	FC	2.08E-16	NA	0	0.375
MC	GB	0.5	NA	0	0.5
MC	CM	-8.33E-17	0.428571	0.07	0.214286
MC	KM	2.08E-16	NA	0	0.3
MC	LR	-1.11E-16	NA	0.58	-3.12E-17
MC	MN	0.247672253	NA	0	0.247672
MU	CL	-0.074074074	NA	0.95	-0.03419
MU	MZ	-0.0726979	NA	0.95	-0.06502
MU	NA1	-0.07274445	NA	0.95	-0.05743
MU	SH	-0.060469996	-0.0083	1	-0.0472
MU	TC	5.11E-17	NA	0	0.007692
MU	AL	-0.104844541	NA	0.95	-0.1018
MU	EC	5.11E-17	0.133333	0	0.03125

MU	FC	-0.074074074	0.133333	0.25	0.027273
MU	GB	-0.074074074	NA	0.7	-0.0125
MU	CM	5.11E-17	0.133333	0	0.047368
MU	KM	-0.074074074	0.133333	0.13	0.05
MU	LR	-0.074074074	0.133333	0.57	-1.78E-17
MU	MN	-0.09964231	NA	0.95	-0.083
CL	MZ	-0.101449275	NA	0.7	-0.10145
CL	NA1	0.065903379	NA	0	0.065903
CL	SH	-0.091279526	0.041448	0.46	0.00752
CL	TC	-8.33E-17	NA	0.25	0.125
CL	AL	-0.044585987	NA	0.7	-0.00836
CL	EC	-8.33E-17	0.25	0.11	0.1
CL	FC	2.08E-16	NA	0	0.166667
CL	GB	0.25	NA	0	0.25
CL	CM	-8.33E-17	0.2	0.07	0.083333
CL	KM	2.08E-16	NA	0	0.125
CL	LR	-0.125	NA	0.7	-0.09091
CL	MN	0.005818786	NA	0	0.005819
MZ	NA1	-0.022258862	NA	0.7	-0.02226
MZ	SH	-0.06565176	-0.03219	1	-0.0412
MZ	TC	0.111111111	NA	0	0.115702
MZ	AL	-0.066666667	NA	0.95	-0.04762
MZ	EC	0.056910569	0.12069	0	0.103789
MZ	FC	0.056910569	NA	0	0.098592
MZ	GB	0.120689655	NA	0	0.12069
MZ	CM	0.056910569	0.111111	0	0.09589
MZ	KM	0.056910569	NA	0	0.087866
MZ	LR	-0.101449275	NA	0.7	-0.05983
MZ	MN	-0.066666667	NA	0.7	-0.06667
NA1	SH	-0.080832527	-0.05056	1	-0.07172
NA1	TC	-0.064073227	NA	0.23	0.111449
NA1	AL	-0.12294094	NA	0.7	-0.06677
NA1	EC	-0.064073227	0.189189	0.1	0.108336
NA1	FC	-0.064073227	NA	0.34	0.020543
NA1	GB	-0.064073227	NA	0.7	-0.06407
NA1	CM	-0.006935843	0.189189	0.05	0.106464
NA1	KM	-0.006935843	NA	0.13	0.047332
NA1	LR	0.065903379	NA	0	0.07526
NA1	MN	-0.12294094	NA	0.7	-0.12294
SH	TC	-0.091279526	0.421451	0.28	0.131809
SH	AL	-0.102557735	0.049753	0.9	-0.06214
SH	EC	-0.066112645	0.415256	0.15	0.163478
SH	FC	-0.091279526	0.204137	0.55	0.007955

SH	GB	-0.091279526	-0.05887	1	-0.06938
SH	CM	-0.017874783	0.421451	0.05	0.164538
SH	KM	-0.091279526	0.134075	0.36	0.026237
SH	LR	-0.091279526	0.204137	0.2	0.060287
SH	MN	-0.102557735	-0.06565	1	-0.09137
TC	AL	-0.157631359	NA	0.95	-0.12575
TC	EC	-0.5	NA	0.72	-0.36364
TC	FC	-8.33E-17	NA	0.32	1.39E-17
TC	GB	-8.33E-17	NA	0.72	-8.33E-17
TC	CM	-0.5	2.08E-16	0.72	-0.28571
TC	KM	-8.33E-17	2.08E-16	0.21	6.25E-17
TC	LR	-8.33E-17	0.25	0.09	0.1
TC	MN	-0.044585987	NA	0.23	0.061528
AL	EC	-0.157631359	0.056911	0.82	-0.08418
AL	FC	-0.044585987	0.056911	0.63	-0.00976
AL	GB	-0.044585987	NA	0.95	-0.04459
AL	CM	-0.157631359	0.056911	0.77	-0.05803
AL	KM	-0.044585987	0.056911	0.45	0.007269
AL	LR	-0.044585987	0.056911	0.34	0.006464
AL	MN	-0.142857143	NA	0.95	-0.09091
EC	FC	-0.2	NA	0.89	-0.09091
EC	GB	-8.33E-17	NA	0.32	1.39E-17
EC	CM	-0.457142857	2.08E-16	0.72	-0.23529
EC	KM	-5.09E-17	2.08E-16	0.16	9.16E-17
EC	LR	-8.33E-17	0.2	0.04	0.083333
EC	MN	-0.044585987	0.111111	0.1	0.060375
FC	GB	2.08E-16	NA	0	2.08E-16
FC	CM	-5.09E-17	2.08E-16	0.16	9.16E-17
FC	KM	2.08E-16	2.08E-16	0	2.08E-16
FC	LR	2.08E-16	0.25	0	0.125
FC	MN	-0.044585987	NA	0.34	0.007269
GB	CM	-8.33E-17	2.08E-16	0.21	6.25E-17
GB	KM	2.08E-16	NA	0	2.08E-16
GB	LR	2.08E-16	NA	0	0.166667
GB	MN	-0.044585987	NA	0.7	-0.04459
CM	KM	-0.2	-8.33E-17	0.99	-0.125
CM	LR	-2.50E-17	0.166667	0.03	0.071429
CM	MN	-0.009762901	0.111111	0.05	0.059683
KM	LR	2.08E-16	0.1875	0	0.1
KM	MN	-0.009762901	NA	0.15	0.024055
LR	MN	0.005818786	NA	0	0.020649

(b) *C. serpentina*

Population1	Population2	Lower bound CI limit	Upper bound CI limit	p-value	Fst
FC	MC	-0.24038	0.186928	0.45	-0.00291
FC	AV	0.09027	0.304348	0	0.194969
FC	MN	-0.19852	0.056911	0.6	-0.06699
FC	SH	-0.11372	NA	0.6	-0.0756
FC	TC	-0.08333	NA	0.17	0.041667
FC	WA	-0.33333	NA	0.46	-0.04762
FC	BR	0	NA	0.25	0.133333
FC	EC	-0.33333	NA	0.93	-0.25
FC	KM	-0.05441	0.166585	0.2	0.067906
FC	MR	-0.03979	0.535429	0.39	0.351874
FC	MU	0	0.037037	0.39	0.022222
FC	TB	-0.08108	-0.03979	1	-0.06024
FC	WR	-0.28099	NA	0.91	-0.21951
MC	AV	0.025168	0.339233	0.01	0.170475
MC	MN	-0.17695	0.089093	0.88	-0.08548
MC	SH	-0.09058	0.244701	0.33	0.05848
MC	TC	-0.08235	-0.04787	0.99	-0.07215
MC	WA	-0.24038	-0.04787	0.99	-0.12318
MC	BR	0.02752	0.191192	0	0.131213
MC	EC	-0.24038	0.174603	0.5	-0.01179
MC	KM	-0.07611	0.009007	0.86	-0.04211
MC	MR	-0.04346	0.460582	0.09	0.254241
MC	MU	-0.10026	0.042245	0.61	-0.00521
MC	TB	-0.04358	0.198998	0.27	0.063561
MC	WR	-0.15904	0.201141	0.33	0.034654
AV	MN	-0.06452	0.0625	0.45	6.43E-17
AV	SH	-0.09234	0.416667	0.17	0.116468
AV	TC	-0.02899	0.270096	0.05	0.115512
AV	WA	0.096774	0.270096	0	0.173114
AV	BR	-0.06619	0.61987	0.09	0.331546
AV	EC	-0.05204	0.304348	0.09	0.147815
AV	KM	-0.04494	0.127389	0.14	0.066148
AV	MR	0.023148	0.119048	0.01	0.084906
AV	MU	-0.17913	0.257485	0.45	0.021021
AV	TB	-0.12903	0.285714	0.4	0.064246
AV	WR	0.095477	0.307136	0.01	0.237968
MN	SH	-0.11231	0.163043	0.49	-0.00232
MN	TC	-0.11511	0.056911	0.93	-0.07916
MN	WA	-0.20863	0.056911	0.93	-0.12069
MN	BR	0.056911	0.223404	0	0.143317
MN	EC	-0.18623	0.056911	0.61	-0.06422
MN	KM	-0.10986	-0.1	0.99	-0.10648

MN	MR	-0.1	0.442197	0.39	0.16954
MN	MU	-0.12338	0.024055	0.93	-0.05691
MN	TB	-0.10753	0.10628	0.69	-0.02462
MN	WR	-0.12974	0.09894	0.56	-0.0235
SH	TC	-0.14142	0.260474	0.25	0.035956
SH	WA	-0.11372	0.260474	0.22	0.051819
SH	BR	0.022003	NA	0	0.160052
SH	EC	-0.11372	NA	0.93	-0.10927
SH	KM	-0.09256	0.242356	0.14	0.090819
SH	MR	0.013978	0.306753	0	0.205863
SH	MU	-0.10026	0.078684	0.88	-0.05653
SH	TB	-0.11458	0.027778	0.88	-0.07578
SH	WR	-0.03583	0.050104	0.51	-0.00768
TC	WA	-0.11111	NA	0.93	-0.1
TC	BR	0.166667	0.416667	0	0.272727
TC	EC	-0.08333	0.166667	0.23	0.037037
TC	KM	-0.14667	-0.03979	0.99	-0.12038
TC	MR	-0.03979	0.253403	0.18	0.114205
TC	MU	-0.16667	0	0.99	-0.1
TC	TB	-0.14252	0.175175	0.46	0.001075
TC	WR	-0.02601	0.202773	0.09	0.09125
WA	BR	0	0.222222	0.03	0.148148
WA	EC	-0.33333	0.166667	0.52	-0.04167
WA	KM	-0.11316	-0.03979	0.99	-0.08748
WA	MR	-0.03979	0.522872	0.18	0.278339
WA	MU	-0.06667	0.037037	0.74	-0.01587
WA	TB	-0.06714	0.175175	0.3	0.041498
WA	WR	-0.25657	0.202773	0.53	-0.00782
BR	EC	-0.02564	NA	0.93	-0.01587
BR	KM	0.067751	0.320895	0.01	0.236055
BR	MR	-0.03979	0.80172	0.07	0.556432
BR	MU	-0.02564	0.533333	0.39	0.252033
BR	TB	-0.08108	0.361217	0.12	0.200094
BR	WR	-0.05263	0.276765	0.32	0.121622
EC	KM	-0.05292	0.159985	0.16	0.06719
EC	MR	-0.03979	0.504108	0.29	0.326195
EC	MU	-0.14286	0.037037	0.66	-0.0101
EC	TB	-0.10484	-0.05629	1	-0.07163
EC	WR	-0.28099	0.03169	0.76	-0.16969
KM	MR	-0.09091	0.219277	0.14	0.105455
KM	MU	-0.12287	1.77E-16	0.95	-0.05898
KM	TB	-0.10435	0.2	0.24	0.063158
KM	WR	-0.00767	0.193702	0.06	0.114216
MR	MU	-0.07986	0.081721	0.58	-0.00127
MR	TB	-2.60E-16	0.208696	0.4	0.123077
MR	WR	-0.01711	0.566919	0.33	0.391925

MU	TB	-0.13427	-0.00267	0.97	-0.08819
MU	WR	-0.00645	0.13324	0.05	0.091621
TB	WR	-0.15859	0.032836	0.76	-0.02698

Supplementary Materials S4: BIC and med scores for DAPC and TESS3R for each dataset.

(a) Best-supported clustering solutions for each dataset.

Species	Dataset	DAPC	TESS3
E. blandingii	Target	4	6
E. blandingii	SNP	1	2
C. serpentina	Target	3	5
C. serpentina	SNP	1	2

(b) *E. blandingii* target gene DAPC (left) and tess3r (right) clustering output.

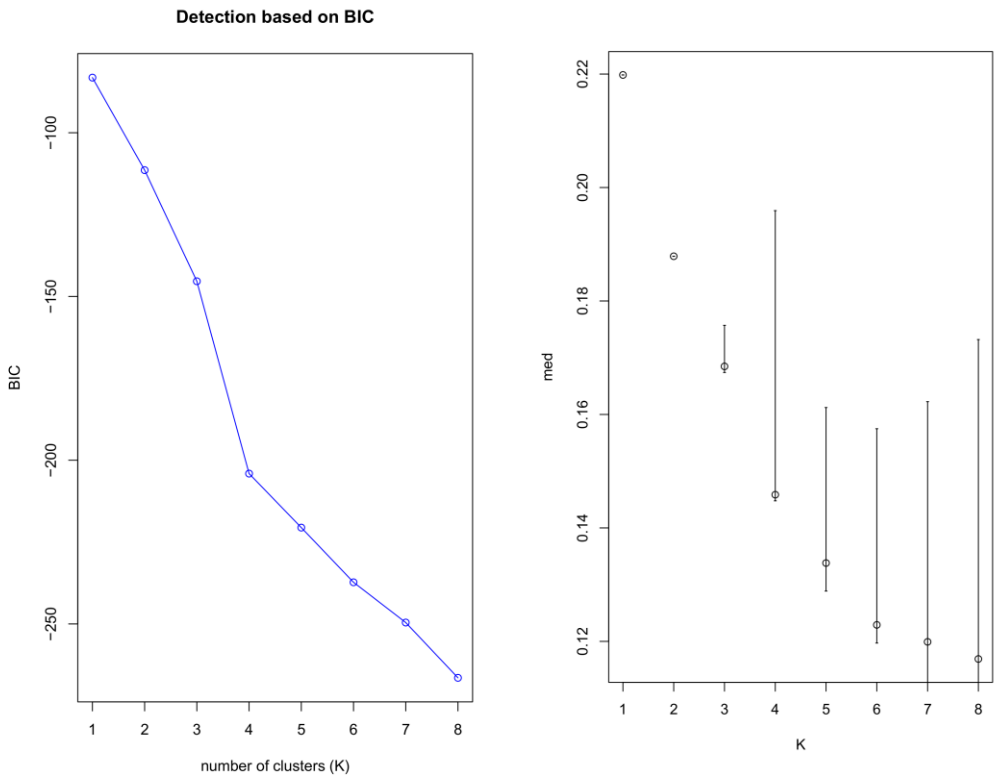

(c) *E. blandingii* SNP DAPC (left) and tess3r (right) clustering output.

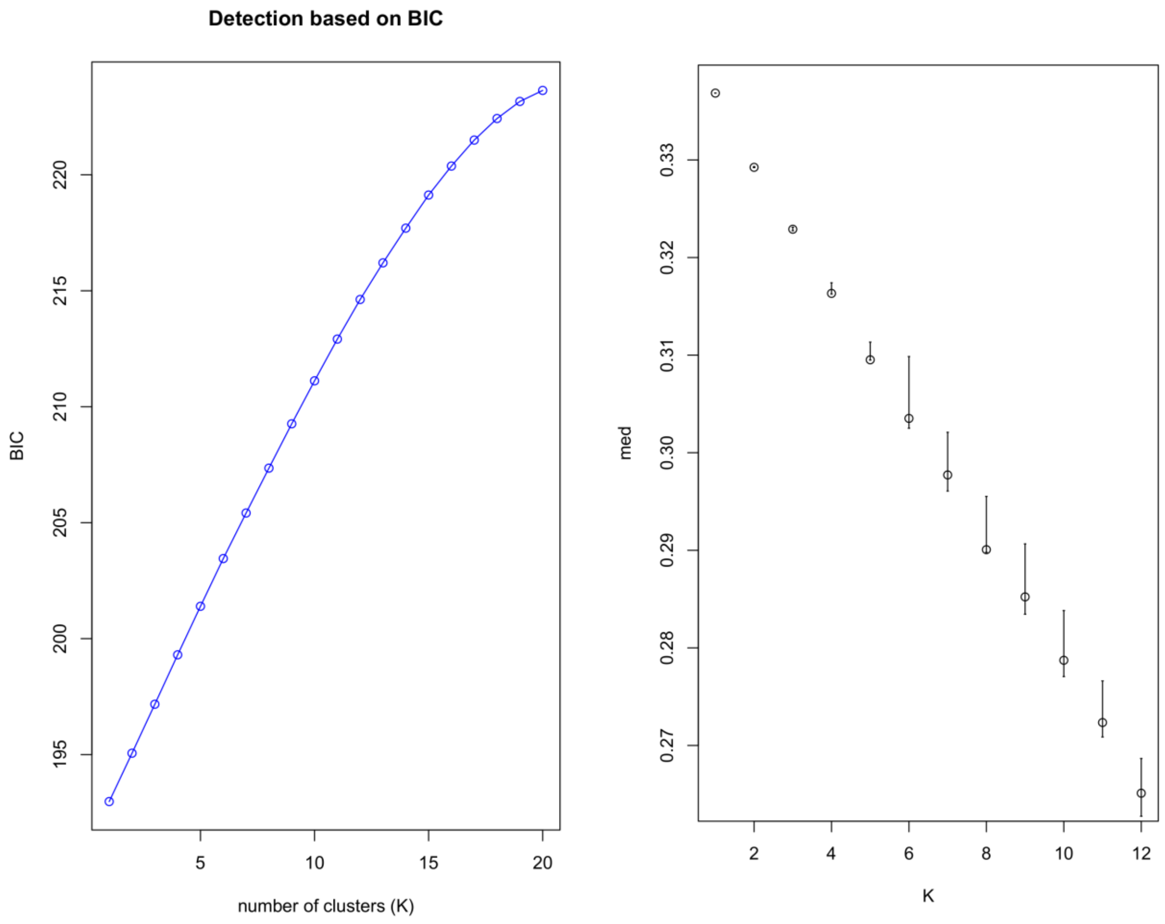

(d) *C. serpentina* target gene DAPC (left) and tess3r (right)

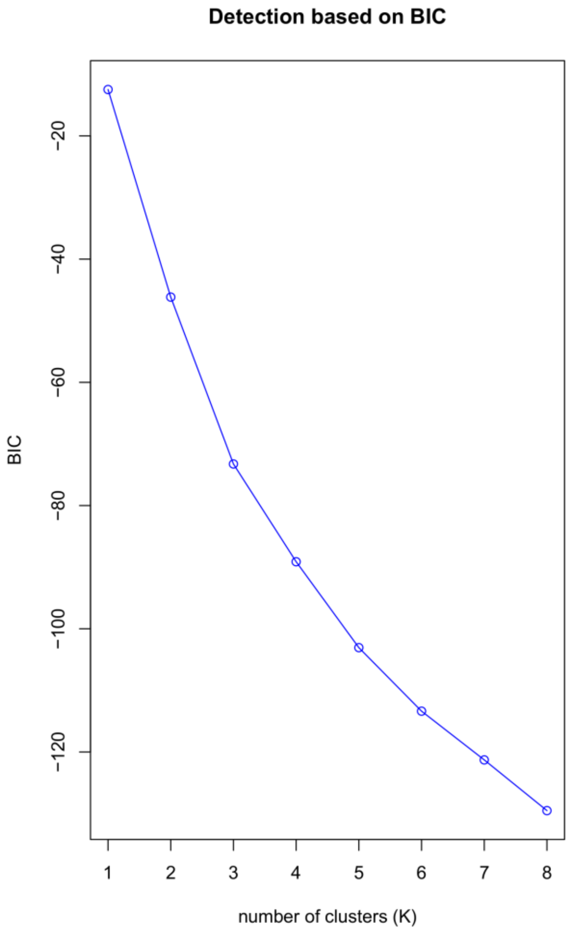

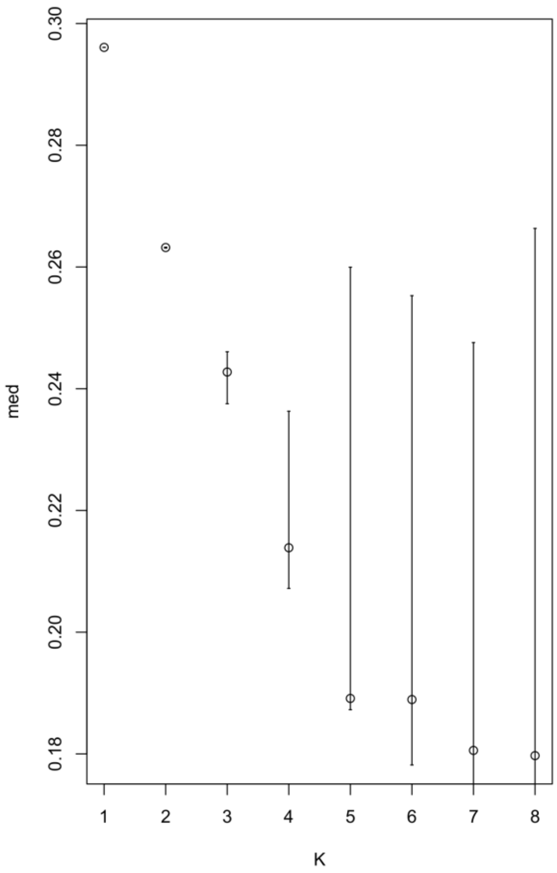

(e) *C. serpentina* SNP DAPC (left) and tess3r (right) clustering output.

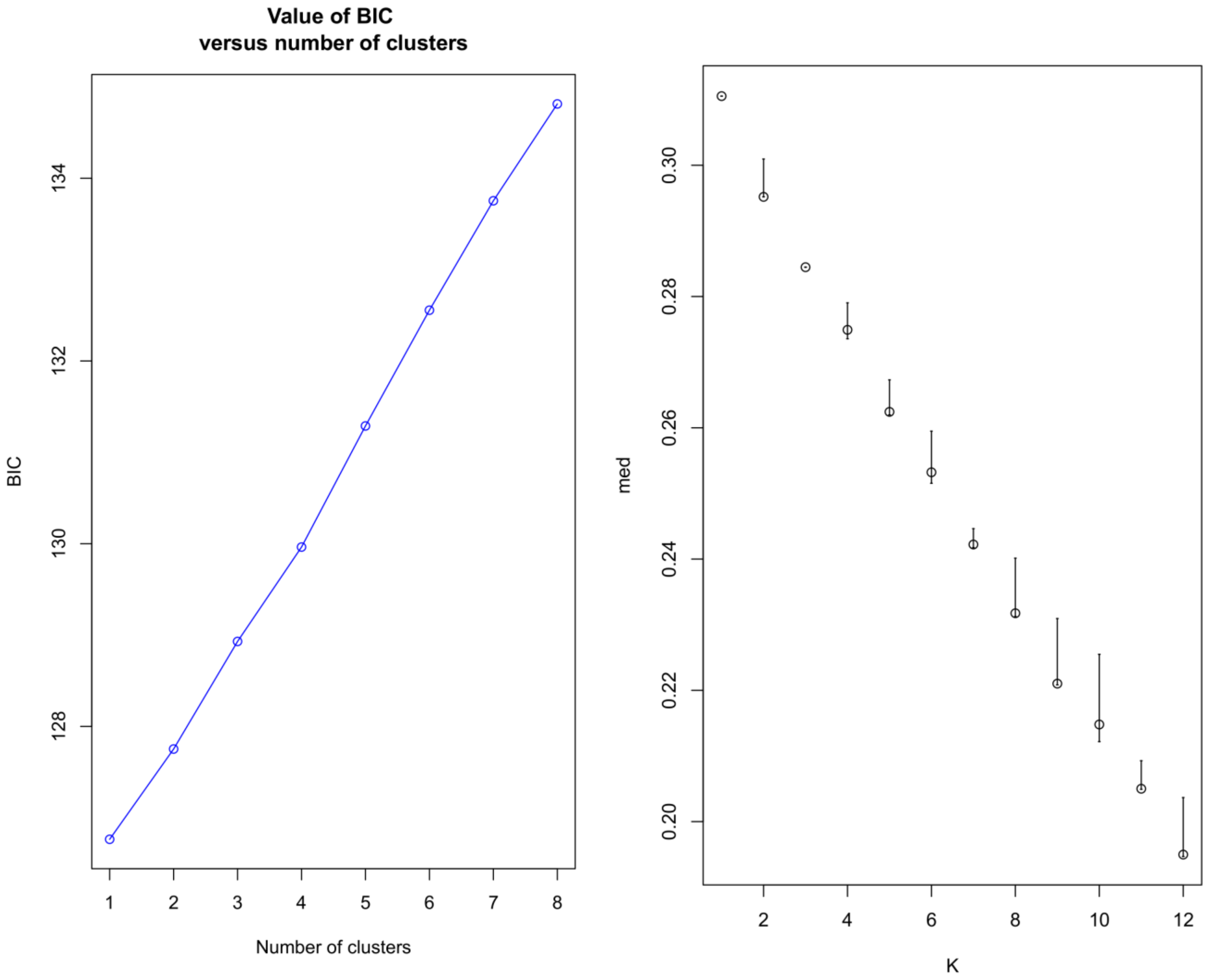

Supplementary Materials S5: Variance partitioning between climate (X1), land cover (X2), and geography (X3) for the full *C. serpentina* target gene dataset.

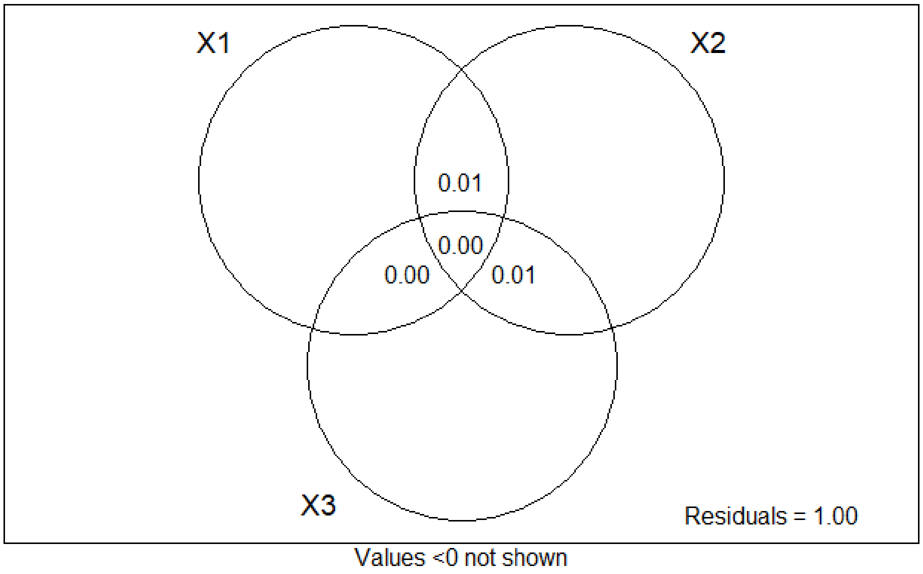

Supplementary Materials S6: On left, relationships between road density and genetic variation in HSPA8 in *E. blandingii* in multivariate space. On right, variance partitioning between climate (X1), land cover (X2), and geography (X3) for the overall *E. blandingii* target gene dataset..

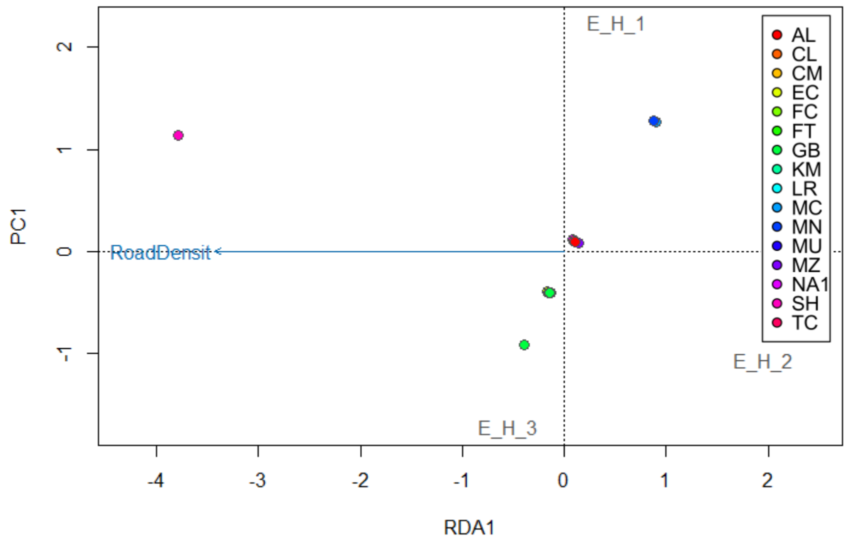

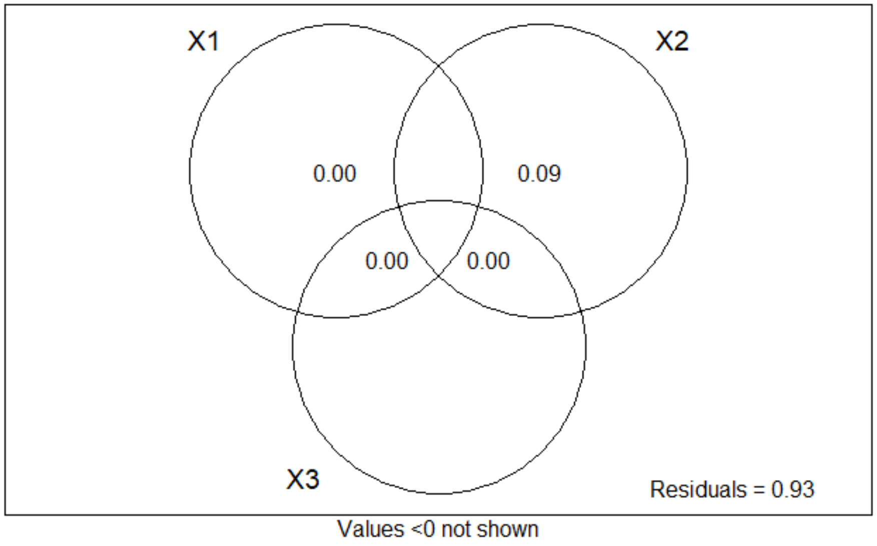

Supplementary Materials S7: (a) Final volumes of reagents in PCR master mixes (all in µL). Each PCR reaction was a total volume of 25 µL and included 3.75 µL of DNA extract. (b) PCR conditions for each primer set. (c) Accessions used for primer creation, all drawn from existing gene-specific sequences from turtles. (d) Table of primer sequences, including the Illumina stem adapter. (e) Qiime2 trimming and truncation settings for each primer set.

(a) PCR reagent volumes

Gene	Polymerase	Water	Buffer	dNTPs	F. primer	R. primer	MgCl	Additive	Polymerase
**CIRBP**	Phusion High-Fidelity	13.75	5	0.5	0.5	0.5	0.5	N/A	0.125
**HSPA8**	Qiagen Taq	15.55	2.5	1	0.5	0.5	1	N/A	0.2
**R35**	Phusion High-Fidelity	13.75	5	0.875	0.5	0.5	0.5	N/A	0.125
**Sox9**	Qiagen Taq	13.55	2.5	1	0.5	0.5	0.5	2.5 µL Q-solution	0.2
**TRPV1**	Phusion High-Fidelity	13.75	5	0.875	0.5	0.5	0.5	N/A	0.125

(b) PCR conditions

Gene	Initial denaturation	Cycles	Denaturation	Annealing	Elongation	Final Elongation
**CIRBP**	30 seconds @ 98ºC	35	10 seconds @ 98 ºC	15 seconds @ 45 ºC	40 seconds @ 72 ºC	5 minutes @ 72 ºC
**HSPA8**	3 minutes @ 94ºC	35	45 seconds @ 94 ºC	45 seconds @ 52 ºC	40 seconds @ 72ºC	10 minutes @ 72 ºC
**R35**	30 seconds @ 98ºC	35	10 seconds @ 98 ºC	15 seconds @ 45 ºC	40 seconds @ 72 ºC	5 minutes @ 72 ºC
**Sox9**	3 minutes @ 94ºC	35	45 seconds @ 94 ºC	45 seconds @ 52 ºC	40 seconds @ 72ºC	10 minutes @ 72 ºC
**TRPV1**	30 seconds @ 98ºC	35	10 seconds @ 98 ºC	15 seconds @ 47 ºC	40 seconds @ 72ºC	5 minutes @ 72 ºC

(c) Accessions used for primer creation

Gene	Accessions used
**CIRBP**	XM_024206191, XM_024206190, XM_024206189, XM_024206188, XM_024206187, XM_024101033, XM_024101031, XM_005302337, XM_024101028, XM_024101027, XM_005302336, XM_014574548. XM_006124966
**HSPA8**	XM_024196662, XM_005303736, KX559320, KX559319, KX559318, KX559317, KX559315, KX559313, KX559311, KX559309, KX559308, KX559306, KX559305, KX559303, KX559301, KX559300, KX559299, KX559298, KX559297, KX559296, KX559294, KX559293, KX559292, KX559290, KX559288, KX559286, KX559285, KX559284, KX559283, KX559282, NM_001286908, KJ581783, XM_007066964, XM_007066963, XM_007066962
**R35**	KF255982, KF255981, KF255980, KF255979, KF255978, KF255977, AJ293980, KP876910, KP876909, KP876908, KP876907, KP876906, KP876905, KP876904, KP876903, KP876902, KP876901, KP876900, KP876899, KP876898, KP876897, KP876896, KP876895, KP876894, KP876893, KP876892, KP876891, KP876890, KP876889, KP876888, KP876887, KP876886, KP876885, KP876884, KP876883, KP876882, KP876881, KP876880, KP876879, KP876878, KP876877, KP876876, KP876875, KP876874, KM411542, KM411541, KM411540, KM411539, KM411538, KM411537, KM411536, KM411535, KM411534, KM411533, KM411532, KM411531, KM411530, KJ582482, KC755167, KC755166, KC755165, KC755164, KC755163, KC755162, KC755161, KC755160, KC755159, KC755158, KC755157, KC755156, KC755155, KC755154, KC755153, KC755152, KC755151, KC755150, KC755149, KC755148, KC755147, KC755146, KC755145, KC755144, KC755143, KC755142, KC755141, KC755140, KC755139, AY434673, AY434672, AY434671, AY434670, AY434669, AY434668, AY434667, AY434666, AY434665, AY434664, AY434663, AY434662, AY434661, AY434660, AY434659, AY434658, AY434657, AY434656, AY434655, AY434654, AY434653, AY434652, AY434651, AY434650, AY434649, AY434648, AY434647, AY434646, AY434645, GU213879, GU213878, GU213877, GU213876, GU213875, GU213874, GU213873, GU213872, GU213871, GU213870, GU213869, GU213868, GU213867, GU213866, GU213865, GU213864, GU213863, GU213862, GU213861, AY905262, AY905261, AY905260, AY905259, AY905258, AY905257, AY905256, AY905255, AY905254, AY905253, AY905252, AY905250, AY905249, AY905248,

	AY905244, AY905242, AY905241, AY905240, AY905238, AY905237, AY905236, AY905235, AY905234, AY905233, AY905232, AY905231, AY905230, AY905229, AY905228, AY905227, AY905226, AY905225, AY905221, AY905220, AY905219, AY905218, AY905217, AY905216, AY905213, AY905211, AY954914, AY954913, AY905251, AY905247, AY905246, AY905245, AY905243, AY905239, AY905224, AY905223, AY905222, AY905215, AY905214, AY905212, HQ260650, HQ260649, HQ260648, AM943826, AJ293981, FN645409, FN645408, FN645407, FN645406, FN645405, FN645404, FN645403, FN645402, FN645401, FN645400, FN645399, KJ475493, LT009772, LT009771, EF587934, EF587933, EF587929, EF587928, EF587924, EF587923, EF587922, EF587921, EF587920, EF587919, LT883268, LT883267, LT883266, LT883265, LT883264, LT883263, LT883262, LT883261, LT883260, LT883259, LT883258, LT883257, KX374285, KX374284, KX374283, KX374282, KX374281, KX374280, KX374279, KX374278, KX374277, KX374276, KX374275, KX374274, KX374273, KX374272, KU883276, KJ913761, FN645398, FN645397, FN645396, FN645395, FN645394, FN645393, FN645392, FN645391, FN645390, FN645389, FN645388, FN645387, FN645386, FN645385, FN645384, FN645383, FN645382, FN645381, FN645380, FN645379, FN645377, KX559331, KX559330, KX559329, KX559328, KX559327, KX559326, KX559325, KX559324, KX559323, KX559322, KX559321, LN834220, LN834276, LN834275, LN834228, LN834227, LN834237, LN834242, LN834277, LN834241, LN834240, LN834239, LN834238, LN834236, LN834235, LN834234, LN834233, LN834232, LN834231, LN834230, LN834229, LN834226, LN834225, LN834224, LN834223, LN834222, LN834221, LN834274, LN834273, LN834272, LN834271, LN834270, LN834269, LN834268, LN834267, LN834266, LN834265, LN834264, LN834263, LN834262, LN834261, LN834260, LN834259, LN834258, LN834257, LN834256, LN834255, LN834254, LN834253, LN834252, LN834251, LN834250, LN834249, LN834248, LN834247, LN834246, LN834245, LN834244, LN834243, LN834219, LN834218, LN834217, LN834216, KR231044, KR231043, KR231042, KR231041, KR231040, KR231039, KR231038, KR231037, KR231036, KR231035, KR231034, KR231033, KR231032, KR231031, KC753147, KC753146, KC753145, KC753144, KC753143, KC753142, KC753141, KC683645, KC683644, KC683643, KC683642, KC683641, KC683640, KC683639, KC683638, KC683637, KC683636, KC683635, KC683634, KC683633, JQ798141, JQ798140, JQ406673, JQ406672, JQ406671, JQ406670, JQ406669, JN860607, JN860606, JN860605, JN860604, JN860603, JN860602, JN860601, JN860600, JN860599, JN860598, JN860597, JN860596, JN860595, JN860594, JN860593, JN860592, JN860591, JN860590, JN860589, JN860588, JN860587, JN860586, JN860585, JN860584, JN860583, JN860582, JN860581, JN860580, JN860579, JN860578, JN860577, JN860576, KC352601, KC352600, KC352599, KC352598, KC352597, KC352596, KC352595, KC352594, KC352593, LM537579, LM537575, LM537572, LM537570, LM537565, LM537583, LM537582, LM537581, LM537580, LM537578, LM537577, LM537576, LM537574, LM537573, LM537571, LM537569,

LM537568, LM537567, LM537566, KJ582959, KJ582958, KJ582957, KJ582956, KJ582955, KJ582954, KJ582953, KJ582952, KJ582951, KJ582950, KJ582949, KJ582948, KJ582947, KJ582946, KJ582945, KJ582944, KJ582943, KJ582942, KJ582941, KJ582940, KJ582939, KJ582938, KJ582937, KJ582936, KJ582935, KJ582934, KJ582933, KJ582932, KJ582931, KJ582930, KJ582929, KJ582928, KJ582927, KJ582926, KJ582925, KJ582924, KJ582923, KJ582922, KJ582921, KJ582920, KJ582919, KJ582918, KJ582917, KJ582916, KJ582915, KJ582914, KJ582913, HE662505, HE662504, HE662503, HE662502, HE662501, HE662500, HE662499, HE662498, HE662497, HE662496, HE662495, HE662494, HE662493, HE662492, HE662491, HE662490, HE662489, HE662488, HE662487, HE662486, HE662485, HE662484, HE662483, HE662482, HE662481, HE662480, HE662479, HE662478, HE662477, HE662476, HE662475, HE662474, HE662473, JN994066, JN994065, JN994064, JN994063, JN994062, JN994061, JN994060, JN994059, JN994058, JN994057, JN994056, JN655668, JN655667, JN655666, JN655665, KC181185, KC181184, KC181183, KC181182, KC181181, KC181180, KC181179, KC181178, KC181177, KC181176, KC181175, KC181174, JN707536, JN707535, JN707534, JN707533, JN707532, JN707531, JN707530, JN707529, JN707528, JN707527, JN707526, JN707525, JN707524, JN707523, JN707522, JN707521, JN707520, JN707519, JN707518, JN707517, JN707516, JN707515, JN707514, JN707513, JN707512, JN707511, JN707510, JN707509, JN707508, JN707507, JN707506, JN707505, JN707504, JN707503, JN707502, JN707501, JN707500, JN707499, JN707498, JN707497, JN707496, JN707495, JN707494, JN707493, JN707492, JN707491, JN707490, HE801911, HE801910, HE801909, HE801908, HE801907, HE801906, HE801905, HE801904, HE801903, HE801902, HE801901, HE801900, HE801899, HE801898, HE801897, HE801896, HE801895, HE801894, HE801893, HE801892, HE801891, HE801890, HE801889, HE801888, HE801887, HE801886, HE801885, HE801884, HE801883, HE801882, HE801881, HE801880, HE801879, HE801878, HE801877, HE801876, HE801875, HE801874, HE801873, HE801872, HE801871, HE801870, HE801869, AY339643, AY339642, AY339641, AY339640, AY339639, AY339638, AY339637, AY339636, AY339635, AY339634, AY339633, AY339632, AY339631, AY339630, AY339629, JN568495, JN568492, JX139081, JX139080, JX139079, JX139078, JQ352077, JQ352076, JQ352075, JQ352074, JQ352073, JQ352072, JQ352071, JQ352070, JQ352069, JQ352068, JN621190, JN621189, JN621188, JN621187, JN621186, JN621185, JN621184, JN621183, JN621182, JN621181, JN621180, JN621179, JN621178, JN621177, JN621176, JN621175, JN621174, JN621173, JN621172, JN621171, JN621170, JN621169, JN621168, JN621167, JN621166, JN621165, JN621164, JN621163, JN621162, JN621161, JN621160, JN621159, JN621158, JN621157, JN621156, JN621155, JN621154, JN621153, JN621152, JN621151, JN621150, JN621149, JN621148, JN621147, JN621146, JN621145, JN621144, JN621143, JF415119, JF415118, JF415117, JF415116, JF415115, JF415114, JF415113, JF415112, JF415111, JF415110, JF415109, JF415108,

	JX067524, JQ596437, JQ596436, JQ596435, JQ596434, JQ596433, JQ596432, JQ596431, JN798059, JN798058, JN798057, JN798056, JN798055, JN798054, JN798053, JN798052, JN798051, JN798050, JN798049, JN798048, JN798047, JN798046, EF011463, EF011462, EF011461, EF011460, EF011459, EF011458, EF011457, EF011456, EF011455, EF011454, EF011453, EF011452, EF011451, EF011450, EF011449, EF011448, EF011447, EF011446, EF011445, EF011444, EF011443, EF011442, EF011441, EF011440, EF011439, EF011438, EF011437, EF011436, EF011435, EF011434, EF011433, EF011432, EF011431, EF011430, EF011429, EF011428, EF011427, EF011426, GQ896251, GQ896250, GQ896249, GQ896248, GQ896247, GQ896246, GQ896245, GQ896244, GQ896243, GQ896242, GQ896241, FJ770712, FJ770711, FJ770710, FJ770709, FJ770708, FJ770707, FJ770706, FJ770705, FJ770704, FJ770703, FJ770702, FJ770701, FJ770700, FJ770699, FJ770698, FJ770697, FJ770696, FJ770695, FJ770694, FJ770693, FJ770692, FJ770691, FJ770690, FJ770689, FJ770688, FJ770687, FJ770686, FJ770685, FJ770684, FJ770683, FJ770682, FJ770681, FJ770680, FJ770679, FJ770678, FJ770677, FJ770676, FJ770675, FJ770674, FJ770673, FJ770672, FJ770671, FJ770670, FJ770669, HE590524, HE590523, HE590522, HE590521, HE590520, HE590519, HE590518, HE590517, HE590516, HE590515, HE590514, HE590513, HE590512, HE590511, HE590510, HE590509, HE590508, HE590507, HE590506, HE590505, HE590504, HE590503, HE590502, HE590501, HE590500, HE590499, HE590498, HE590497, HE590496, HE590495, HQ442388, HQ442387, HQ442386, HQ442385, HQ442384, HQ442383, HQ442382, HQ442381, HQ442380, HQ442379, HQ442378, HQ442377, HQ442376, HQ442375, HQ442374, HQ442373, HQ442372, HQ442371, HQ442370, HQ266664, FR717085, FR717084, FR717083, FR717082, FR717081, FR717080, FR717079, FR717078, FR717077, FR717076, FR717075, FR717074, FR717073, FR717072, FR717071, FR717070, FR717069, FR717068, FR717067, FR717066, FR717065, FR717064, FR717063, FR717062, FR717061, FR717060, FR717059, FR717058, FR717057, FR717056, FR717055, FR717054, FR717053, FR717052, FR717051, FR717050, FR717049, FR717048, FR717047, FR717046, GQ259475, GQ259474, GQ259473, GQ259472, GQ259471, GQ259470, GQ259469, GQ259468, GQ259467, GQ259466, GQ259465, GQ259464, GQ259463, GQ259462, GQ259461, GQ259460, GQ259459, DQ661020, DQ649464, DQ649463, DQ649461, DQ649460, DQ649459, DQ649458, DQ649457, DQ649456, DQ649455, FM877773, FM877772, FM877771, FN645410, FN645378, GU085692, GU085691, GU085690, GU085689, GU085688, GU085687, GU085686, GU085685, GU085684, GU085683, GU085682, GU064311, GU064310, GU064309, GU064308, GU064307, GU064306, GU064305, GU064304, GU064303, GU064302, GU064301, GU064300, GU064299, GU064298, GU064297, GU064296, GU064295, GU064294, GU064293, GU064292, GU064291, GU064290, GU064289, GU064288, GU064287, GU064286, GU064285, GU064284, GU064283, GU064282, GU064281, GU064280, GU064279, GU064278, GU064277, GU064276, GU064275, GU064274, GU064273, GU064272, GU064271, GU064270, GU064269, GU064268, GU064267, GU064266,

	GU064265, GU064264, GU064263, GU064262, GU064261, FJ230877, FJ230870, FJ230863, FJ230856, FJ039995, FJ039988, FJ039981, FJ039974, FJ039967, FJ039960, FJ039959, FJ039952, FJ039945, FJ039938, FJ039931, FJ039924, FJ039917, FJ039910, FJ009031, FJ009024, EU277645, EU277644, EU277643, EU277642, EU277641, EU277640, EU277639, EU277638, EU277637, EU277636, EU277635, EU277634, EU277633, EU277632, EU277631, EU277630, EU277629, EU277628, EU277627, EU277626, EU277625, EU277624, EU277623, EU277622, EU277621, EU277620, EU277619, EU277618, EU277617, EU277616, EU277615, EU277614, EU277613, EU277612, EU277611, EU277610, EU277609, EU277608, EU277607, EU277606, EU277605, EU277604, EU277603, EU277602, EU277601, EU277600, EU277599, EU277598, EU277597, EU277596, EU277595, EU277594, EU169879, EU169878, EU169877, EU169876, EU169875, DQ386681, DQ386680, DQ386679, DQ386678, DQ386677, DQ386676, DQ386675, DQ386674, DQ386673, DQ386672, DQ386671, DQ386670, DQ386669, DQ386668, DQ386667, DQ386666, DQ386665, DQ386664, DQ386663, DQ386662, DQ386661, DQ386660, DQ386659, DQ386658, DQ386657, DQ386656, DQ386655, DQ386654, DQ386653, DQ386652, AY742463, AY742462, AY742461, AY742460, AY742459, AY742458, AY742457, AY742456, AY742455, EU787218, EU787217, EU787216, EU787215, EU787214, EU787213, EU787212, EU787211, EU787210, EU787209, EU787208, EU787207, EU787206, EU787205, EU787204, EU787203, EU787202, EU787201, EU787200, EU787199, EU787198, EU787197, EU787196, EU787195, EU787194, EU787193, EU787192, EU787191, EU787190, EU787189, EU787188, EU787187, EU787186, EU787185, EU787184, EU787183, EU787182, EU787181, EU787180, EU787179, EU787178, EU787177, EU787176, EU787175, EU787174, EU787173, EU787172, EU787171, EU787170, EU787169, EU787168, EU787167, EU787166, EU787165, EU787164, EU787163, EU787162, EU787161, EU787160, EU787159, FJ234440, AM943849, AM943848, AM943847, AM943846, AM943845, AM943844, AM943843, AM931708, AM931707, AM931706, AM931705, AM931704, AM931703, AM931702, AM931701, AM931700, AM931699, AM931698, AM931697, AM931696, AM931695, AM931694, AM931623, EF587932, EF587931, EF587930, EF587927, EF587926, EF587925, EF587917, EF587916, EF587915, DQ649462, DQ649454, KJ482686, KJ482685, HQ020481, HQ020480, HQ020479, HQ020478, HQ020477, EU119956, EU119955, EU119954, EU119953, EU119952, EU119951, EU119950, EU119949, EU119948, EU119947, EU119946, EU119945, EU119944, EU119943, EU119942, EU119941, EU119940, EU119939, EU119938, EU119937, EU119936, EU119935, EU119934, EU119933, EU119932, EU119931, EU119930, EU119929, EU119928, EU119927, EU119926, EU119925, EU119924, EU119923, EU119922, EU119921, EU119920, EU119919, EU119918, EU119917, EU119916, EU119915, EU119914, EU119913, EU119912, EU119911, EU119910, EU119909, EU119908, EU119907, EU119906, EU119905, EU119904, EU119903, EU119902, EU119901, EU119900, EU119899, DQ529131, DQ529130, DQ529129, DQ529128, DQ529127, DQ529126, DQ529125, DQ529124,

	DQ529123, DQ529122, DQ529121, DQ529120, DQ529119, DQ529118, AY259595, AY259594, AY259593, AY259592, AY259591, AY259590, AY259589, AY259588, AY259587, AY259586, AY259585, AY259584, AY259583, AY259582, AY259581, AY259580, AY259579, AY259578, AY259577, AY259576, AY259575, AY259574, AY259573, AY259572, AY259571, KC484758, JQ950727, JQ950726, JQ950725, JQ950724, JQ950723, JQ950722, HQ260657, HQ260656, HQ260655, HQ260654, HQ260653, HQ260652, HQ260651
**Sox9**	XM_024196757, XM_005282966, KU821117, EU233431, EF524567, AY168554, AY168537, AF335422, XM_014576568, XM_007056058, EU268286, GQ258676, EU914820
**TRPV1**	XM_024211218, XM_024105926, XM_005298178, XM_007057655

(d) Primer sequences

Gene	Primer sequence (F)	Primer sequence (R)
**CIRBP**	ACACTCTTTCCCTACACGACGCTCTTCCGATCT/**TTGATGGGCGTCAGATTAGA**	GTGACTGGAGTTCAGACGTGTGCTCTTCCGATCT/**TGAGCCTTACAGTATTTCCA**
**HSPA8**	ACACTCTTTCCCTACACGACGCTCTTCCGATCT/**CCTTCCTTGGATGTCTGAGC**	GTGACTGGAGTTCAGACGTGTGCTCTTCCGATCT/**GGTGTGGAAGAGTAGTATGCTAAGC**
**R35**	ACACTCTTTCCCTACACGACGCTCTTCCGATCT/**GATGCAATGTATGGGAAG**	GTGACTGGAGTTCAGACGTGTGCTCTTCCGATCT/**TCTCTCTCTATTTCTCMGAATG**
**Sox9**	ACACTCTTTCCCTACACGACGCTCTTCCGATCT/**AAGGACCATCCCGACTACAAG**	GTGACTGGAGTTCAGACGTGTGCTCTTCCGATCT/**GCCCTTCTYGCTTCAGGTC**
**TRPV1**	ACACTCTTTCCCTACACGACGCTCTTCCGATCT/**GGAAAGATGAGGAAACTTGGC**	GTGACTGGAGTTCAGACGTGTGCTCTTCCGATCT/**TCATTCAGATCACTTGTGTCAC**

(e) Trimming and truncation settings

Gene	trim setting	trunc setting
**CIRBP**	0	200
**HSPA8**	0	200
**R35**	0	0
**Sox9**	0	0
**TRPV1**	0	0
